# Concurrent Amplification of Ferroptosis and Immune System Activation Via Nanomedicine‐Mediated Radiosensitization for Triple‐Negative Breast Cancer Therapy

**DOI:** 10.1002/advs.202407833

**Published:** 2024-12-25

**Authors:** Reyida Aishajiang, Zhongshan Liu, Yuan Liang, Pengye Du, Yi Wei, Xiqian Zhuo, Shuyu Liu, Pengpeng Lei, Tiejun Wang, Duo Yu

**Affiliations:** ^1^ Department of Radiotherapy The Second Hospital of Jilin University Changchun 130022 China; ^2^ State Key Laboratory of Rare Earth Resource Utilization Changchun Institute of Applied Chemistry Chinese Academy of Sciences Changchun Jilin 130022 China; ^3^ School of Applied Chemistry and Engineering University of Science and Technology of China Hefei Anhui 230026 China

**Keywords:** cGAS‐STING pathway, ferroptosis, nanomedicine, radiation therapy, ROS

## Abstract

Radiation therapy (RT) is one of the core therapies for current cancer management. However, the emergence of radioresistance has become a major cause of radiotherapy failure and disease progression. Therefore, overcoming radioresistance to achieve highly effective treatment for refractory tumors is significant yet challenging. Here, pH‐responsive DSPE‐PEoz modified hollow Bi_2_Se_3_‐RSL3/diABZi (DP‐HBN/RA) nanomedicine is designed as a radiation sensitizer for efficient treatment of triple‐negative breast cancer by simultaneously amplifying ferroptosis and immune system activation. DP‐HBN/RA can efficiently concentrate X‐ray radiation energy inside the tumor, thereby promoting precise ionizing radiation exposure in tumor cells to produce large amounts of reactive oxygen species (ROS), leading to lipid peroxidation‐induced ferroptosis. Meanwhile, ferroptotic cell death is intensified through the inactivation of GPX4 by RSL3 released from DP‐HBN/RA to acidic conditions in the tumor microenvironment. Additionally, DP‐HBN/RA enhances RT efficacy to exacerbate unrepairable DNA damage and release DNA fragments that activate the cGAS‐STING signal pathway, evoking a systematic immune response. Ingeniously, the released diABZi reinforces cGAS‐STING activation to boost the immunology antitumor effect. This work links the induction of ferroptosis and the initiation of systematic immune response to achieve highly effective tumor suppression, which opens up new avenues for future treatments of refractory tumors.

## Graphical abstract

DP‐HBN/RA simultaneously enhances tumor ferroptosis and immune response in TNBC radiotherapy. DP‐HBN/RA efficiently absorbs X‐rays, leading to elevated ROS levels and modulation of ferroptosis‐related enzymes to inhibit tumor growth. Concurrently, DP‐HBN/RA releases RSL3 in response to the acidic tumor microenvironment, thereby reducing tumor radioresistance and enhancing ferroptosis. Additionally, RT‐induced DNA damage activates systematic immune response, and the released diABZi enhances the cGAS‐STING pathway activation to promote the immune response.

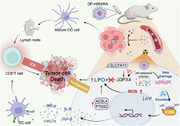

## 1 Introduction

Triple‐negative breast cancer (TNBC) is a particularly aggressive phenotype of breast cancer, characterized by high metastasis and “cold” tumor immune microenvironment, remains low targeting efficacy, and poor prognosis.^[^ ^1^, ^2^, ^3^, ^4^ ^]^ Radiation therapy (RT) is one of the powerful treatments for TNBC, utilizing X‐ray to break DNA strands via accumulation of reactive oxygen species (ROS) to produce DNA fragments and interrupt diverse cellular signal pathways to cell death.^[^ ^5^, ^6^ ^]^ However, many TNBC patients initially respond to RT while advanced TNBC partially develops resistance to RT as residual survived tumor cells cause inevitable recurrence and metastasis, ultimately leading to RT failure.^[^ ^7^ ^]^ To address this issue, it's urgent to integrate reliable biomarkers to enhance the effectiveness of RT in TNBC. Fortunately, among various biomarkers for prolonged TNBC survival, ferroptosis‐related metabolites have attracted increased attention to improve RT response in TNBC. In addition, a combination of immune adjuvants presented greater therapeutic efficacy in RT than monotherapy for TNBC.^[^ ^2^ ^]^ Moreover, one approach has shown promise which is the use of high‐Z element‐containing nanomedicines to sensitize tumor cells to RT.^[^ ^8^, ^9^ ^]^ Therefore, targeting ferroptosis and systemic immunity by combining small molecule compounds that are independent of apoptotic cell death to bypass primary resistance mechanisms, with a high‐Z element containing nanomedicines is highly desirable for enhancing the efficacy of RT in TNBC.

Ferroptosis is a form of regulated cell death distinct from apoptosis and necrosis, characterized by iron dependency and lipid peroxidation (LPO).^[^ ^10^, ^11^ ^]^ It is important for cancer because many tumor cells exhibit altered iron metabolism and oxidative stress management, potentially making them more susceptible to ferroptosis.^[^ ^2^, ^12^, ^13^, ^14^, ^15^ ^]^ Thus, ferroptosis‐targeted therapy has been recognized as a potential strategy for suppressing tumor growth. Recent research also revealed the potent ferroptosis sensitivity of TNBC as well. According to the latest analysis in different region of TNBC tissue, GPX4, and SLC7A11 were proved to upregulated in the large region of tumor‐related to diminish of LPO, indicating a potent biomarker that benefits precise treatment.^[^ ^16^ ^]^ Besides, another crucial factor such as ACSL4, related LPO was also reported to be highly expressed in subtypes of breast cancer and regulate ferroptosis sensitivity.^[^ ^17^ ^]^ Moreover, recent studies proved that GPX4‐targeted ferroptosis inducers (FINs) including RAS‐selective lethal compounds 3 (RSL3), could inhibit TNBC proliferation and growth of tumors.^[^ ^2^, ^18^, ^19^, ^20^ ^]^ Considering the therapeutic efficiency of both ferroptosis and RT is dependent on ROS, RT could be an ideal antitumor mediator to induce ferroptosis. Mechanistically, RT disrupts the dynamic equilibrium of ROS, causing damage to biological molecules and inducing LPO to trigger ferroptosis. A recent study also revealed that ferroptosis‐inducing enzymes such as ACSL4 and PTGS2 could be upregulated following irradiation to promote ferroptosis in tumors.^[^ ^21^ ^]^ However, adaptive responses to RT, such as the elevation of ferroptosis inhibitors like GPX4 and SLC7A11, might contribute to radioresistance by diminishing the effects of ferroptosis. Therefore, combining RT with ferroptosis inducers (FINs) might enhance therapeutic efficacy. Hopefully, the latest cohort study found that GPX4 was elevated in TNBC, and increased expression of ACSL4 and inhibiting GPX4 might sensitize cells to ferroptosis.^[^ ^22^, ^23^ ^]^ Moreover, Zeng et al. applied high‐Z element Gold‐based MOF resulted in significant improvement in RT efficacy in TNBC.^[^ ^24^ ^]^ These studies provoke the idea that combining GPX4‐targeted FINs with high‐Z element‐based nanomaterials as RT sensitizers as an antitumor strategy in TNBC cells with insufficient ferroptosis holds promise for improving treatment outcomes and overcoming radioresistance.

Recently, the integration of immunotherapy with RT can significantly impact the overall survival of cancer patients by enhancing therapeutic efficacy. One key pathway involving immune responses to DNA damage is the cGAS‐STING signaling pathway, which plays a crucial role in inducing immunogenic cell death (ICD). The cGAS acts as a cytosolic DNA sensor that binds to DNA fragments and triggers the production of the second messenger cGAMP. This activation of cGAS by tumor‐derived DNA leads to the activation of the cGAS‐STING pathway, which in turn stimulates systematic antitumor immunity.^[^ ^2^ ^]^ This is particularly relevant in TNBC, where stimulating the cGAS‐STING signalling pathway has been shown to increase therapeutic efficacy in TNBC.^[^ ^25^, ^26^, ^27^ ^]^ Besides, when excessive ROS are generated, intracellular DNA can be released from mitochondria and sensed by cGAS, resulting in the production of type I interferons and inflammatory cytokines.^[^ ^28^ ^]^ A previous study demonstrated that combining immunotherapy with RT‐induced ferroptosis can enhance the expression of cGAS, PTGS2, and CD8^+^ cells, leading to improved disease‐free survival rates in cancer treatment.^[^ ^29^ ^]^ Furthermore, studies have shown that GPX4 deficiency could activate the cGAS‐STING signaling pathway by increasing ROS and DNA damage, leading to suppression of tumorigenesis in TNBC.^[^ ^30^ ^]^ Therefore, employing high‐Z element containing nanomedicines to enhance the efficacy of RT and increase DNA damage to trigger the cGAS‐STING signal pathway is a robust antitumor immune response strategy.

**Figure 1:**
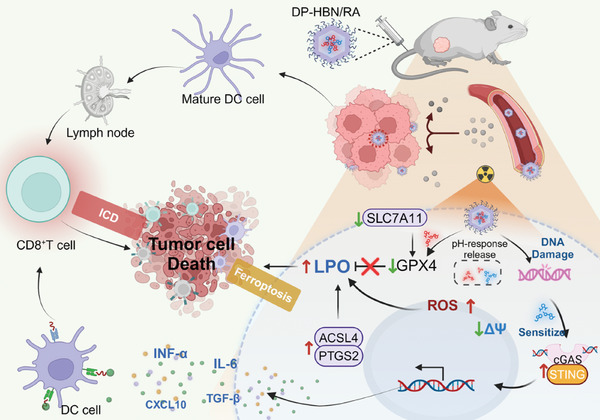
Schematic illustration of the therapeutic mechanism of DP‐HBN/RA with concurrent amplification of ferroptosis and immune system activation ability. The Figure was created with BioRender.com.

In this work, RSL3 and STING agonist diABZi‐compound 3 (diABZi) were co‐loaded into hollow Bi_2_Se_3_ nanoparticles (HBN) to achieve concurrent amplification of tumor cell ferroptosis and systemic immune response, which were finally modified by pH‐responsive 1,2‐Distearoyl‐sn‐glycero‐3‐phosphoethanolamine‐Poly(2‐ethyl‐2‐oxazoline) (DSPE‐Peoz) to obtain DP‐HBN/RA. The antitumor mechanism of DP‐HBN/RA is shown in **Figure** **1**. DP‐HBN/RA has the ability to absorb X‐rays efficiently and induce tumor ferroptosis through the enhanced ROS level to upgrade LPO levels and modulate ferroptosis‐related enzymes. This process leads to significant inhibition of tumor growth. Owing to the pH‐sensitive properties of DSPE‐PEoz, RSL3, and diABZi can be released specifically in response to an acidic tumor microenvironment. Along with the released RSL3, a GPX4‐targeted FINs inhibits the adaptive upregulation of the antioxidant enzyme GPX4, thereby reducing potent tumor radioresistance and enhancing ferroptosis to suppress tumor growth. Additionally, DP‐HBN/RA can promote precise ionizing radiation exposure in tumor cells to enhance DNA damage and generate DNA fragments, which activate the cGAS‐STING pathway. This pathway plays a critical role in initiating an immune response against tumor cells. Furthermore, released diABZi increases the sensitivity of the cGAS sensor to double‐stranded DNA (dsDNA), reinforces cGAS‐STING activation to elevate the level of immunology cytokines (IFN‐β, TNF‐α, CXCL10, and IL‐6) and CD4^+^/CD8^+^ infiltration, thereby evoking an efficient systematic immune response. Finally, the antitumor effect of DP‐HBN/RA with concurrent amplification of ferroptosis and immune system activation was demonstrated in detail.

## 2 Result and Discussion

### 2.1 Preparation and Characterization

**Figure 2:**
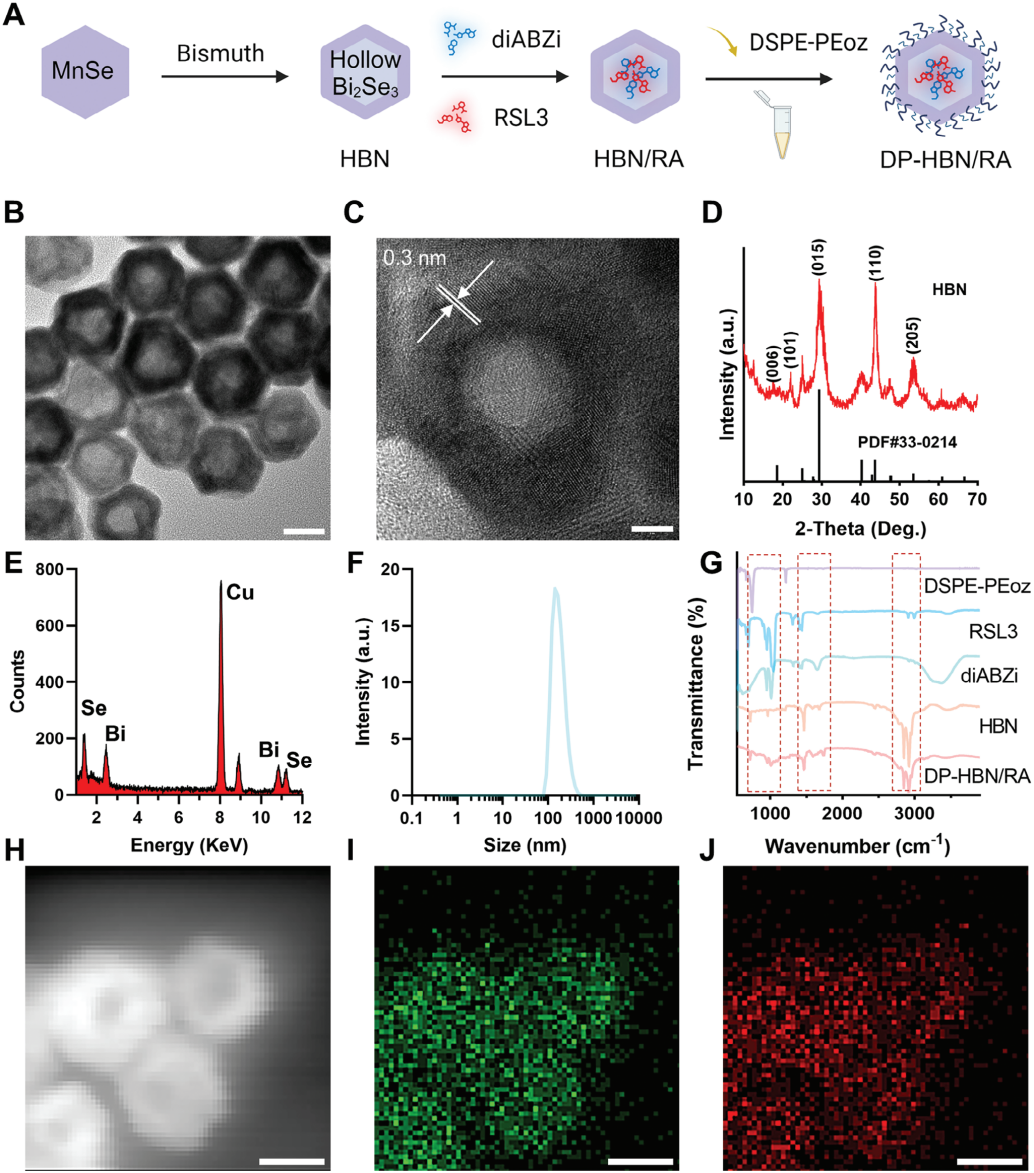
A) The synthesis process of DP‐HBN/RA. The Figure was created with BioRender.com. B) TEM image, scale bar = 20 nm. C) HRTEM image, scale bar = 5 nm. D) XRD pattern of HBN. E) EDS spectrum of HBN. F) DLS of DP‐HBN/RA. G) FTIR spectra of DP‐HBN/RA, HBN, DSPE‐PEOZ, RSL3 and diABZi. H–J) Elemental mapping images of HBN, scale bar = 20 nm.

HBN was rationally synthesized through a cation exchange strategy with a nanoscale Kirkendall effect. Briefly, utilizing MnSe nanoparticles as a template. Subsequently, a bismuth source was injected under heating to obtain HBN.^[^ ^31^ ^]^ Then RSL3 and diABZi were co‐loaded into HBN and finally modified with DSPE‐PEoz to obtain DP‐HBN/RA (**Figure** **2**A). As visualized by the transmission electron microscopic (TEM) images, HBN exhibits an average size of ≈34.26 nm (Figure 2B). High‐resolution TEM (HRTEM) presents the fringe spacing of HBN as 0.3 nm, corresponding to the (205) crystal plane of the standard hexagonal phase of Bi_2_Se_3_ (Figure 2C). Selected area electron diffraction shows the polycrystalline nature of HBN (Figure S1, Supporting Information). As expected, X‐ray photoelectron spectroscopy (XPS) and X‐ray powder diffraction (XRD) data confirm the successful synthesization of HBN, of which the XRD results match the standard card PDF#33‐0214 (Figure 2D; Figure S2, Supporting Information). The presence of Bi and Se are determined by an Energy dispersive spectrometer (EDS) (Figure 2E). Moreover, the hydrodynamic diameter of DP‐HBN/RA is approximately 176 nm (Figure 2F), and after co‐incubation with other human simulated solutions for 24 h, dynamic light scattering (DLS) results show no significant difference compared to the group in water (Figure S3, Supporting Information), indicating the stable character of DP‐HBN/RA. The release of RSL3 and diABZi significantly increased with decreasing pH, indicating that the pH‐sensitive DSPE‐PEoz successfully enabled DP‐HBN/RA to release the drugs in a pH‐dependent manner (Figure S4, Supporting Information). Observation of changes in zeta potential, and Fourier transform infrared spectroscopy (FTIR) reveals characteristic functional groups of DEPC‐PEoz, diABZi, and RSL3 in DP‐HBN/RA, suggesting successful loading of drugs and modification of DSPE‐PEoz (Figure 2G; Figure S5, Supporting Information). Further, elemental mapping of HBN also provides evidence the presence of Bi and Se elements (Figure 2H–J).

### 2.2 In Vitro and In Vivo CT Imaging and Biocompatibility Analysis

**Figure 3:**
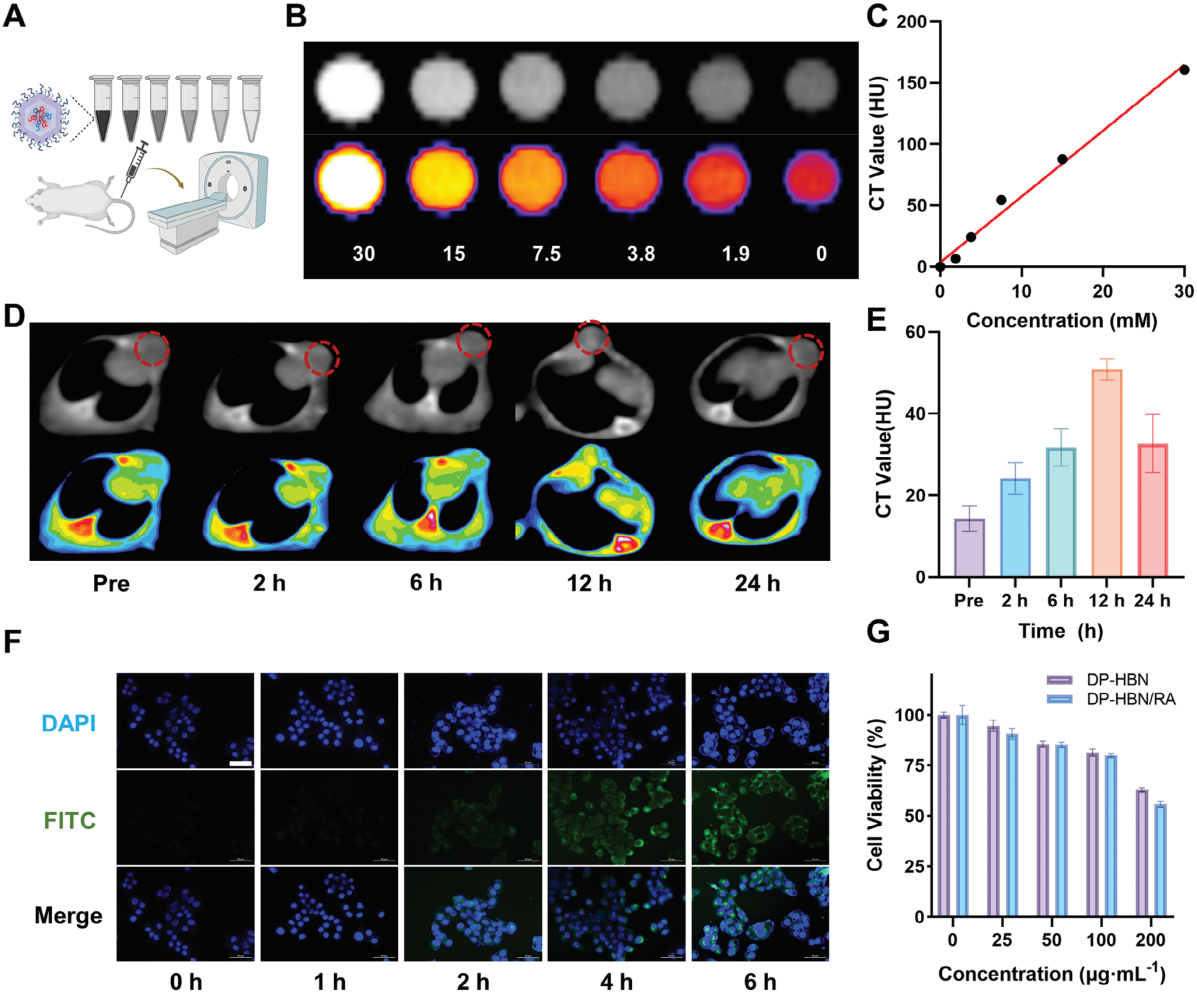
A) Illustration of CT imaging process. The Figure was created with BioRender.com. B,C) In vitro CT images and associated CT value (HU) of DP‐HBN/RA at different concentrations. D,E) In vivo CT images and associated CT value of the 4T1‐bearing mice after intravenous injection of DP‐HBN/RA at timed intervals (tumor, circle). F) Cellular uptake analysis of DP‐HBN/RA in 4T1 cells, scale bar = 50 µm. G) Cytotoxicity of DP‐HBN and DP‐HBN/RA with different concentrations.

Bismuth (Bi) possesses a high atomic number with a large X‐ray attenuation coefficient at 100 keV, making it a promising CT contrast agent.^[^ ^32^ ^]^ We first studied the in vitro CT imaging of DP‐HBN/RA, and the results in **Figure** **3**B,C show that the Hounsfield unit (HU) of CT signal linearly increases with the concentration of DP‐HBN/RA (the average X‐ray attenuation coefficient is 5.37 HU mM). In vivo, CT imaging reveals an initial increase in CT signals on the tumor side for the first 12 h after tail vein injection, followed by a decrease (Figure 3D,E). This suggests that DP‐HBN/RA could serve as an effective CT image enhancer and implies that injecting 12 h prior to RT might be the best timing to yield optimal therapeutic efficacy. The hemolysis experiments demonstrate excellent blood compatibility, with values below the critical 5% hemolysis threshold (Figure S6, Supporting Information), indicating that DP‐HBN/RA is a highly biocompatible nanomedicine. Furthermore, the cellular uptake capacity was investigated using FITC‐labeled DP‐HBN/RA. The observed time‐dependent increase in fluorescence intensity in 4T1 cells confirms the effective internalization of DP‐HBN/RA (Figure 3F; Figure S7, Supporting Information). Additionally, we observed that as the concentration increased, DP‐HBN/RA exhibited a significant cytotoxic effect. This could be attributed to the loaded RSL3 and diABZi triggering ferroptosis and immune response, while cell viability in DECP‐PEoz‐hollow Bi_2_Se_3_ (DP‐HBN) was relatively high (Figure 3G). To investigate drug combination synergy, we used SynergyFinder, which is a computational tool to predict and analyze drug combination synergy used in cancer research.^[^ ^33^, ^34^ ^]^ The Bliss synergy plot shows that RSL3/diABZi ratio at (10:1) could achieve the best synergetic effect with a Bliss Score of 21.04 (Figure S8, Supporting Information). To further investigate the impact of tumor ferroptosis and the cGAS‐STING pathway under single‐agent loading conditions, we conducted western immunoblotting (WB) and Elisa experiments (Figures S9 and S10, Supporting Information). These analyses revealed that the activation effects of RSL3 or diABZi administered individually were significantly less effective than those observed with simultaneous dual‐agent loading. This finding reinforces the notion of a synergistic effect when RSL3 and diABZi are co‐loaded. Consequently, in our subsequent experiments, we focused exclusively on dual‐loaded nanomedicines for a more comprehensive analysis. According to tolerable dose testing, 5 mg kg^−1^ is determined as the maximum tolerable dose (Figures S11 and S12 and Table S1, Supporting Information). To evaluate in vivo toxicity, we conducted hematoxylin and eosin (H&E) staining on major organs of mice injected with DP‐HBN/RA after 28 d. The H&E staining results in the DP‐HBN/RA group show no significant difference compared to the control group, which supports the evidence of biocompatibility of DP‐HBN/RA (Figure S13, Supporting Information).

### 2.3 Assessment of Radiosensitization Ability

**Figure 4:**
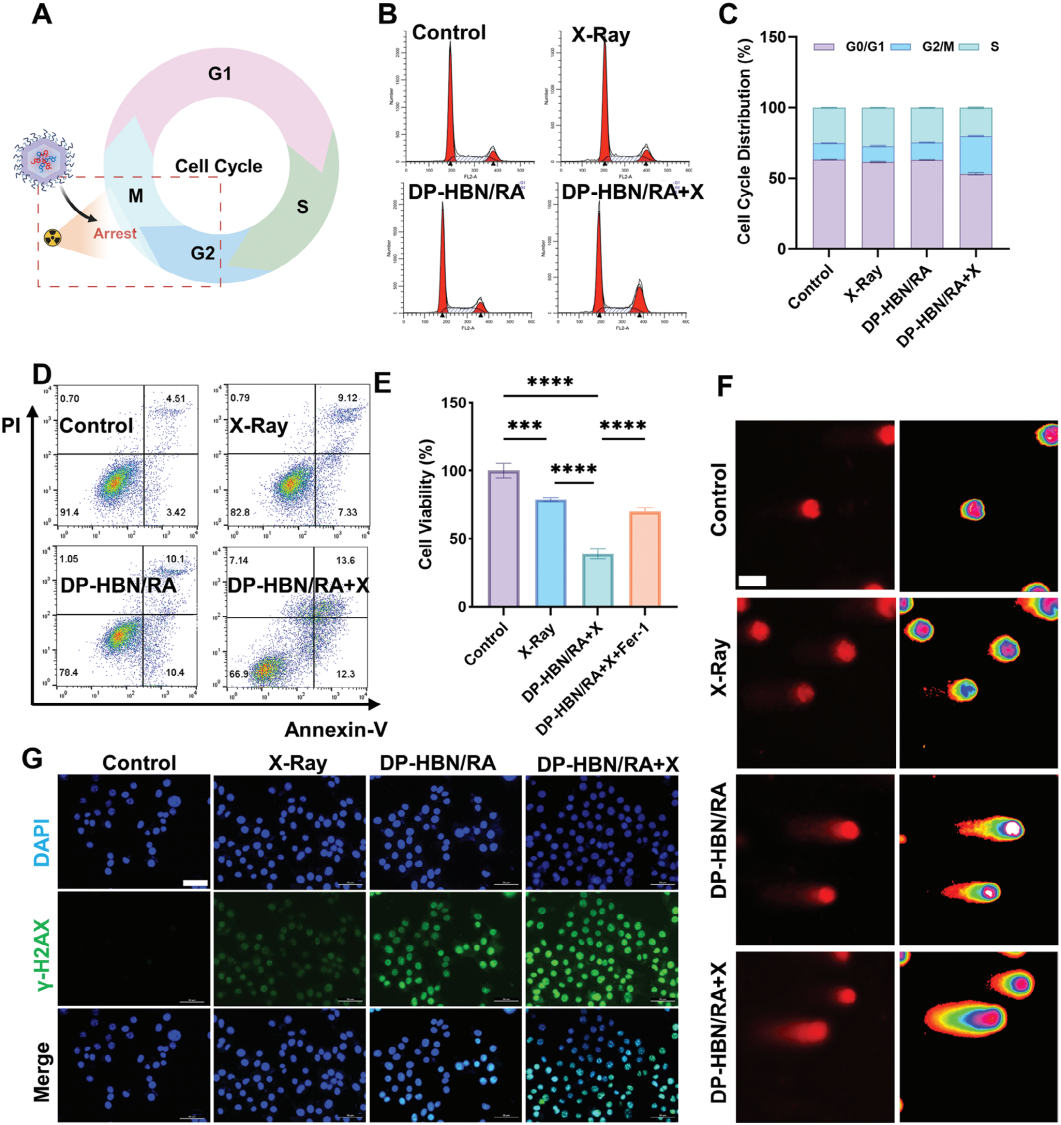
A) Illustration of cell cycle under RT. The Figure was created with BioRender.com. B,C) Cell cycle analysis and percentage in 4T1 cells with different treatments. D) Flow cytometric analysis of 4T1 cells with different treatments. E) Cytotoxicity of DP‐HBN/RA with Fer‐1 (2 µM) under different treatments.(ANOVA; ^***^ *p* < 0.001 and ^****^ *p* < 0.0001) F) Commet assay with different treatments, scale bar = 50 µm. G) DNA damage with γ‐H2AX immunofluorescence with different treatments, scale bar = 50 µm. Data are presented as mean ± standard deviation. (ANOVA; ^***^ *p* < 0.001 and ^****^ *p* < 0.0001).

DNA double‐strand breaks (DSBs) induced by X‐ray irradiation are the most severe incidents that could influence multiple biomolecular systems, including chromosomal rearrangements, cell cycle arrest, and cell death.^[^ ^35^, ^36^ ^]^ The cell cycle plays a crucial role in determining radiosensitivity by regulating DNA replication, which influences cell proliferation. Most cells exhibit radioresistance to radiation during the S phase and sensitivity during the G2/M phase.^[^ ^37^ ^]^ Therefore, modulation of the cell cycle could be employed to enhance the efficacy of RT. In this study, the cell cycle of DP‐HBN/RA was predominantly distributed at the G2/M phase with post‐irradiation. This indicates that DP‐HBN/RA may sensitize RT by arresting the G2/M phase (**Figure** **4**B,C). Moreover, RT damages DNA, resulting in the termination of cell proliferation and playing a significant role in tumor‐suppressing efficacy. Cell Counting Kit‐8 (CCK‐8) and flow cytometry analysis were employed to assess the inhibitory effect of DP‐HBN/RA on cell growth. As presented in Figure 4D,E and Figure S14 (Supporting Information), DP‐HBN/RA enhances RT efficacy in inhibiting cell growth. However, when co‐cultured with Fer‐1 (a ferroptosis inhibitor), there was a notable rescue of decreased cell growth, especially in the group with DP‐HBN/RA combined RT (DP‐HBN/RA+X). Clonogenic survival assay is considered one of the standard ways of detecting resistance to RT. DP‐HBN/RA also presented significant clonal inhibition after radiation. Furthermore, Fer‐1 restored the clonogenic survival that was reduced by exposure to DP‐HBN/RA and RT (Figure S15, Supporting Information). These findings strongly suggest that DP‐HBN/RA could enhance the antitumor effect by increasing cell death and suppressing RT resistance, with ferroptosis playing a significant role. The loaded RSL3 greatly increases ferroptotic cell death to enhance RT efficacy. To be noted, RT can lead to DNA strand breaks, some of which could be partially repaired.^[^ ^38^ ^]^ Therefore, the ability to repair DNA damage is an important factor contributing to radioresistance. The comet assay is a sensitive method for detecting DNA damage, the greater the damage, the longer the tail will be.^[^ ^39^ ^]^ As shown in Figure 4F, the DP‐HBN/RA+X group presents the longest comet tail, indicating the most significant DNA damage effect. Consistent with this result, the marker γ‐H2AX (a DNA damage detection method) also reveals greater DNA damage in DP‐HBN/RA+X (Figure 4G). Migration ability is often associated with distant metastasis. Here, the scratch assay was employed, a common method to measure cell migration, particularly in tumor studies.^[^ ^40^ ^]^ As shown in Figure S16 (Supporting Information), DP‐HBN/RA has a notable anti‐migration effect, especially when combined with RT. These results highlight the significant antitumor capability of DP‐HBN/RA under RT conditions by generating large amounts of unrepairable DNA damage, arresting the G2/M phase, and inhibiting cell proliferation and migration.

### 2.4 Enhance RT Efficacy Through Amplifying Ferroptosis

**Figure 5:**
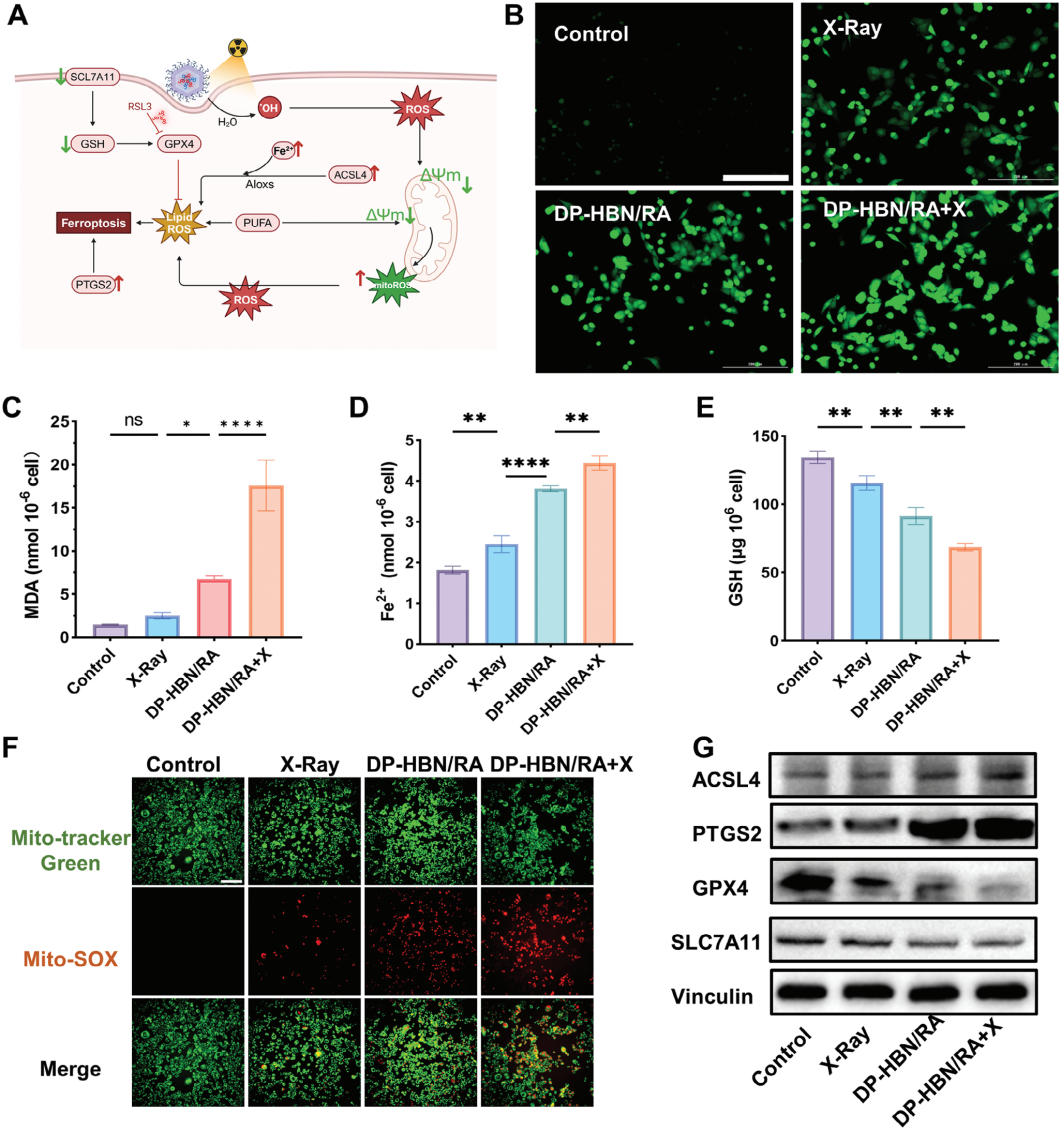
A) Illustration of DP‐HBN/RA enhances RT efficacy by amplifying ferroptosis. The Figure was created with BioRender.com. B) Total ROS accumulation in 4T1 with different treatments, scale bar = 200 µm. C) MDA level in 4T1 cells with different treatments. D) Fe^2+^ level in 4T1 cells with different treatments. E) GSH level in 4T1 cells with different treatments. F) MitoROS accumulation in 4T1 cells with different treatments, scale bar = 200 µm. G) Western blotting analysis of ACSL4, PTGS2, SLC7A11, and GPX4 expression in 4T1 cells with different treatments. Data are presented as mean ± standard deviation. (ANOVA; ns for no significance, ^**^ *p* < 0.01, and ^****^ *p* < 0.0001).

Ferroptosis is caused by excessive oxidative damage, wherein ROS can react with the polyunsaturated fatty acids (PUFAs) of lipid membranes and induce LPO.^[^ ^10^, ^41^ ^]^ DCFH2‐DA was employed to quantify the ROS level in 4T1 cells after different treatments. As expected, RT increased the total ROS in 4T1 cells, and the involvement of DP‐HBN/RA could further enhance the ROS level, especially when combined with radiation. (**Figure** **5**B). Since malondialdehyde (MDA) is one of the products of LPO, it is important to trigger ferroptosis.^[^ ^42^ ^]^ MDA levels were tested 24 h after radiation in 4T1 cells and presented elevated levels after being treated with DP‐HBN/RA. The increased MDA suggests that DP‐HBN/RA+X enhances LPO, supporting the evidence that DP‐HBN/RA could enhance RT efficacy by boosting oxidative stress to trigger ferroptosis via LPO. Besides, RSL3 and diABZi amplify ferroptotic and immune effects, potentially contributing to excessive oxidative damage (Figure 5C). Glutathione (GSH) and Fe^2+^ play roles in LPO. Briefly, Fe^2+^ requires oxidation to Fe^3+^ for lipoxygenase activity, while GSH levels help defend against LPO.^[^ ^43^ ^]^ Our results showed that DP‐HBN/RA led to an increase in Fe^2+^ and a decrease in GSH levels after RT, indicating that DP‐HBN/RA could enhance RT efficacy by elevating Fe^2+^ concentration and reducing GSH to promote ferroptosis (Figure 5D and E). A previous study revealed that declined cysteine could hyperpolarize mitochondria, leading to LPO accumulation and mitochondrial ferroptosis.^[^ ^2^ ^]^ Here, JC‐1 was applied to quantify mitochondrial membrane potential, and found that the DP‐HBN/RA+X group had the most significant impact (Figure S17, Supporting Information), demonstrating its potential ability to modulate mitochondrial dysfunction. As is well known that mitochondria are the primary source of ROS.^[^ ^44^ ^]^ Using Mito‐SOX, a mitochondrial‐targeted ROS probe, DP‐HBN/RA presented the best effect on inducing mitochondrial ROS (MitoROS) after irradiation (Figure 5F). There were marker genes and proteins encompassing key regulators of ferroptosis, such as ferroptosis inducers ACSL4 and PTGS2, and inhibitors GPX4 and SLC7A11.^[^ ^42^ ^]^ The DP‐HBN/RA+X group significantly increased ACSL4 and PTGS2 expression while decreasing GPX4 and SLC7A11 levels (Figure 5G). Together, these results manifested that DP‐HBN/RA could enhance RT efficacy by promoting ferroptosis through multiple mechanisms. On the one hand, DP‐HBN/RA enhanced X‐ray energy absorption, generating more ROS, which contributed to LPO. Excessive ROS hyperpolarized mitochondrial membrane potential, increasing MitoROS and promoting ferroptosis. On the other hand, RSL3 in DP‐HBN/RA inhibited the adaptive increase in GPX4 during radiation in 4T1 cells, diminishing radioresistance to increase ferroptotic cell death.

### 2.5 Enhance RT Efficacy Through Activating Immune Response

**Figure 6:**
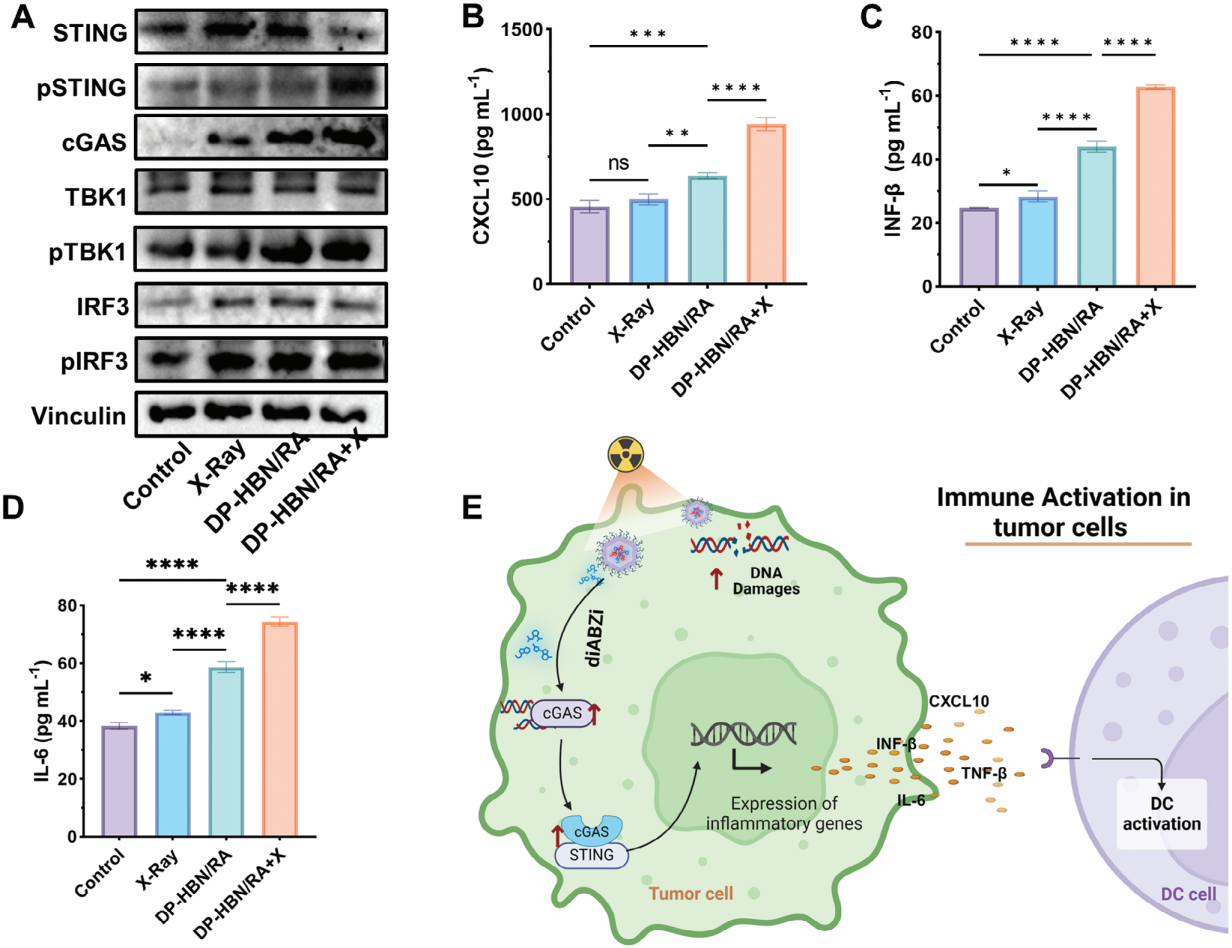
A) Western blotting analysis of pSTING, STING, pTBK1, TBK1, pIRF3, IRF3, and cGAS expression in 4T1 cells with different treatments. B) Expression of CXCL10 C), INF‐β), and D) IL‐6 B in 4T1 supernatant with different treatments. E) Illustration of DP‐HBN/RA amplify cGAS‐STING pathway in tumor cells. The Figure was created with BioRender.com. Data are presented as mean ± standard deviation. (ANOVA; ns for no significance, ^**^ *p* < 0.01, ^***^ *p* < 0.001, and ^****^ *p* < 0.0001).

The cGAS‐STING signal pathway is a critical innate immune pathway that is activated by DNA‐damaging treatments. This activation triggers the cGAS‐TBK1‐IRF3 cascade, which stimulates distinct immune effectors that are responsive to tumorigenesis and metastasis.^[^ ^2^ ^]^ Notably, the expression levels of proteins related to the cGAS‐STING pathway, including pSTING, pTBK1, pIRF3, and cGAS, were markedly increased in 4T1 cells in the DP‐HBN/RA+X group (**Figure** **6**A). These results indicate that DP‐HBN/RA enhances the activation of the cGAS‐STING signal pathway in RT, which may elicit a stronger immune response in tumor cells. To be noted that DNA‐damaging treatments could initiate the cGAS‐STING cascade to release immunological cytokines to evoke a systematic immune response.^[^ ^2^ ^]^ To further explore the immune response ability of DP‐HBN/RA, IFN‐α, TNF‐α, CXCL10, and IL‐6 were detected in 4T1 supernatants after different treatments. Obviously, the concentrations of CXCL10, IFN‐β, IL‐6, and TNF‐α were significantly elevated in the DP‐HBN/RA+X group (Figure 6B–D; Figure S18, Supporting Information). This demonstrates that DP‐HBN/RA has the ability to strengthen the systematic immune response by boosting the secretion of immunological cytokines in 4T1 cells. Taken together, DP‐HBN/RA sensitizes RT by producing and releasing abundant damaged DNA fragments that strengthen cGAS activation and subsequently secrete inflammatory cytokines, of which diABZi could contribute to reinforced cGAS‐STING signal pathway activation by sensitizing cGAS to DNA fragments, which paves the potent way for a potent evoking of a robust systematic immune response.

### 2.6 In Vivo Antitumor Efficacy

**Figure 7:**
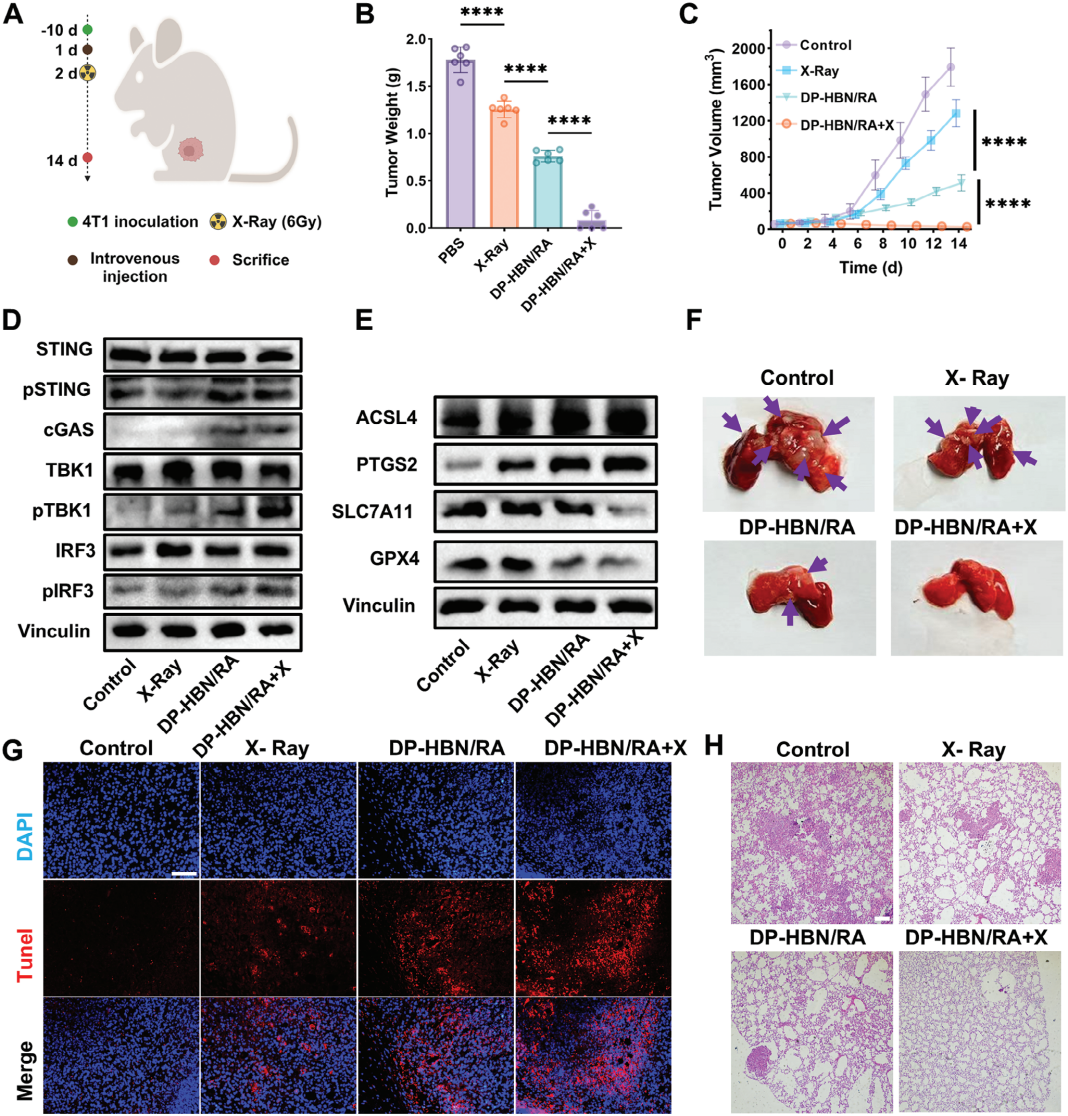
A) Treatment schema of In vivo antitumor experiments. The Figure was created with BioRender.com. B) Weight of 4T1 xenograft tumors with different treatments. C) Volume of 4T1 xenograft tumors with different time points after different treatments. D) Western blotting analysis of STING‐related proteins in 4T1 xenograft tumors. E) Western blotting analysis of ferroptosis‐related proteins in 4T1 xenograft tumors. F) The photo of lung metastasis at 14 d different treatments. G) TUNEL staining images of 4T1 xenograft tumors at 14 d with different treatments, scale bar = 50 µm. H) H&E staining of lung metastasis sections at 14 d with different treatments, scale bar = 200 µm. Data are presented as mean ± standard deviation. (ANOVA; **** *p* < 0.0001).

To determine the antitumor ability of DP‐HBN/RA in vivo, DP‐HBN/RA was intravenously injected into 4T1 tumor‐bearing mice. Compared to the control group, the growth of tumors was inhibited in the RT group; however, the therapeutic efficacy was not satisfactory. Notably, the inhibitory effect of tumor growth was increased in the DP‐HBN/RA group, particularly in the DP‐HBN/RA+X group presenting a significant tumor‐suppressing effect (**Figure** **7**B,C). Moreover, during the treatment, the application of DP‐HBN/RA had no obvious changes in body weight compared to the control group (Figure S19, Supporting Information). This result supports the evidence that DP‐HBN/RA is a good biocompatible nanomedicine in vivo. In addition, we also investigated the antitumor mechanism of DP‐HBN/RA using western blotting. As expected, the ferroptosis and cGAS‐STING upregulated proteins in the DP‐HBN/RA+X group were higher than those in the RT alone group (Figure 7D,E), respectively, further confirming that DP‐HBN/RA concurrently amplifies ferroptosis and cGAS‐STING signal pathway to suppress tumor growth. We also characterized the antitumor immunity of DP‐HBN/RA by flow cytometry analysis. Compared to the RT group, the involvement of DP‐HBN/RA significantly increased CD80^+^ and CD86^+^ levels in lymph nodes after radiation, indicating maturation of DC cells, which could subsequently evoke a systematic immune response by triggering cytotoxic T cells to kill tumor cells, respectively. Notably, the percentage of CD8^+^ and CD4^+^ were significantly elevated in the DP‐HBN/RA+X group, indicating that DP‐HBN/RA reversed the “cold” tumor immune microenvironment by amplifying the cGAS‐STING signal pathway to sensitize RT efficacy (Figure S20, Supporting Information). We also employed TUNEL, immunofluorescence staining (IHC), and H&E to confirm the antitumor efficacy of DP‐HBN/RA. Increased fluorescence in TNUEL staining assays and increased γH2AX marker IHC verified that DP‐HBN/RA+X enhanced RT efficacy by increasing excessive DNA damage, which is also consistent with broken tissue structure in H&E stating images. In addition, PTGS2 and GPX4 markers in IHC showed the same tendency as western blotting (Figure 7G; Figure S21, Supporting Information). Inspired by the promising results of DP‐HBN/RA in combination with RT for primary tumor treatment and immune modulation, we developed a lung metastasis model to evaluate the DP‐HBN/RA efficacy in preventing tumor invasion and metastasis. As illustrated in Figure 7F,H and Figure S22 (Supporting Information), histological analysis of lung tissues via H&E staining revealed a substantial presence of metastatic nodules in the control group, whereas the DP‐HBN/RA+X group exhibited almost no detectable metastatic nodules. Additionally, flow cytometric analysis of lung tissue demonstrated a significant increase in CD8+ T cell infiltration (Figure S23, Supporting Information). These findings indicate that DP‐HBN/RA effectively inhibits both primary tumor growth and metastatic spread. Moreover, the H&E staining of major organs in the DP‐HBN/RA group shows no significant difference compared to the control group, which indicates evidence of biocompatibility of DP‐HBN/RA (Figure S24, Supporting Information). These results further suggest that RT is less effective in inducing ferroptosis and immune response when used alone, while combined DP‐HBN/RA significantly suppresses tumor growth in TNBC by inducing excessive ferroptosis and amplifying antitumor immune response.

## 3 Conclusion

In conclusion, we successfully designed a nanomedicine that concurrently enhances ferroptosis and immune system activation to improve the efficiency of RT against triple‐negative breast cancer. The pronounced ferroptosis effect on tumor cells can be attributable to the DP‐HBN/RA radiosensitizing effect, which produces a large amount of ROS that leads to excessive LPO, and RSL3 mitigates potential RT resistance by reinforcing ferroptotic cell death. Besides, DP‐HBN/RA efficiently focuses X‐ray radiation energy in tumors, which intensifies DNA damage that robustly stimulates the cGAS‐STING signal pathway to evoke a strong systematic immune response. Meanwhile, diABZi strengthens cGAS sensitivity to DNA fragments that contribute to cGAS‐STING activation. This work paves the way for the development of simultaneous amplification of ferroptosis and systematic immune response in enhancing antitumor therapy for refractory tumors.

## 4 Experimental Section

### Statistic Analisis

All results were expressed as the mean ± standard deviation. One‐way analysis of variance (ANOVA) was employed for statistical significance analysis. The statistical significance was indicated by ns for no significance, ^*^ for *p* < 0.05, ^**^ for *p* < 0.01, ^***^ for *p* < 0.001, and ^****^ for *p* < 0.0001. Data were analyzed by GraphPad Prism9.5.

### Animal Ethics

Balb/c female mice (3–4 weeks) were purchased from Changsheng Biotechnology Co. Ltd. (320 700 000 055 905). All of the animal experiments were approved by the Institutional Animal Care and Use Committee of Changchun Institute of Applied Chemistry (IACUC) (Grant no. 20 240 096).

## Conflict of Interest

The authors declare no conflict of interest.

## Supporting information



Supporting Information

## Data Availability

The data that support the findings of this study are available in the supplementary material of this article.
