# Supplementary material for: Concurrent Amplification of Ferroptosis and Immune System Activation Via Nanomedicine‐Mediated Radiosensitization for Triple‐Negative Breast Cancer Therapy
Source: Adv Sci (Weinh). 2024 Dec 25;12(7):2407833. doi: 10.1002/advs.202407833 (PMC11831504; doi:10.1002/advs.202407833)
Supplement: Supplementary file 1 — Supporting Information [file ADVS-12-2407833-s001.docx]

Supporting Information

Concurrent Amplification of Ferroptosis and Immune System Activation via Nanomedicine-Mediated Radiation Sensitization for Triple-Negative Breast Cancer Therapy

*Reyida Aishajiang, Zhongshan Liu, Yuan Liang, Pengye Du, Yi Wei, Xiqian Zhuo, Shuyu Liu, Pengpeng Lei*, Tiejun Wang*, and Duo Yu**

R. Aishajiang, Z. S. Liu, X. Q. Zhuo, T. J. Wang, D. Yu

The Second Hospital of Jilin University

Department of Radiotherapy, Changchun 130022, China

E-mail: m13943016598@163.com, yuduo@jlu.edu.cn (D. Yu).

Y. Liang, P. Y. Du, Y. Wei, S. Y. Liu, and P. P. Lei

State Key Laboratory of Rare Earth Resource Utilization, Changchun Institute of Applied Chemistry, Chinese Academy of Sciences, Changchun, Jilin 130022, China

E-mail: leipp@ciac.ac.cn (P. P. Lei)

Y. Liang, P. Y. Du, and S. Y. Liu

School of Applied Chemistry and Engineering, University of Science and Technology of China, Hefei, Anhui 230026, China

**Keywords:** ferroptosis; cGAS-STING pathway; ROS; nanomedicine; radiation therapy

**Experimental Section**

**Materials**

Manganese chloride tetrahydrate (MnCl_2_·4H_2_O), selenium power (Se, AR), and 1-octadecene (ODE) were purchased from Aladdin. Oleylamine (OLA) and bismuth neodecanoate was obtained from Sigma-Aldrich. DSPE-Peoz was purchased form Ruixi Biological Technology (Xi’an, China). 1,2-dipalmitoyl-sn-glycero-3-phosphocholine (DPPC) and Cholesterol were purchased from Aladdin. diABZi was purchased from Selleck. RSL3 was purchased from MedChemExpress. Cell Counting Kit-8 (CCK-8)-Assay kit was obtained from Bioss. MDA and GSH assay kit were purchased from Solarbio. Iron assay kit was obtained from Elabsicence. JC-1 Mitochondrial membrane potential assay kit was purchased from Beyotime. Annexin V-FITC apoptosis assay kit was purchased from Meilunbio.

**Synthesis of HBN**

0.2 mM MnCl_2_·H_2_O was added to 1 mL OLA and 9 mL ODE, degassed at 140 ℃, and slowly heated to 280 ℃ in A flask. At the same time, 0.2 mM Se source in 1ml OLA was degassed at 120 ℃ for at least 1 hour and then heated to 250 ℃ for 2 h and cooled to 80 ℃ in B flask. Subsequently, the mixture in B was injected into A flask and then heated to 300 ℃ for 10 min to obtain MnSe. After cooling down to 50 ℃, 1ml ODE with 0.04 mM bismuth neodecanoate was injected in to flask A. Finally, the mixture was heated at 180 ℃ for 30 min to obtain hollow Bi_2_Se_3_.

**Synthesis of DP-HBN/RA**

First prepare DSPE-PEoz solution by mixing cholesterol and DPPC with chloroform. Then added RSL3 and diABZi into 5mL HBN solution with hexyl hydride under ultrasonic for 10 min, and added DSPE-PEoz solution. The optimal RSL3/diABZi feeding ratio was determined at 4:1 (w/w) to achieve an equivalent loading ratio of 10:1for final products. Subsequently, the mixture was stirring for night at room temperature. Finally, rotary evaporator was employed to remove excess solvent, then added PBS with ultrasonic to dissolve DP-HBN/RA.

**Characterization**

Transmission electron microscopic (TEM) images were processed under FEI Tecnai G2 S-Twin at 200 kV. X-Ray photoelectron spectroscopy (XPS) was recorded on a VG ESCALAB MK II electron spectrometer. X-Ray powder diffraction (XRD) was tested with D8 ADVANCE (Bruker). UV-Vis-NIR absorption spectra detected with Shimadzu UV-3600. Zeta potential and dynamic light scattering were carried out with Zetasizer Nano Z (Malvern). X-ray irradiation was conducted with X-ray bioirradiator (Precision X-Ray) with 1.0 Gy/min. The RSL3 drug feeding amount was 53.3%, while diABZi was feeding amount of 21.4% (drug loading capacity with 12% for RSL3 and 1.2% for diABZi.).Therelease behavior of RSL3 and diABZi were calculated with optimal density by Multimode Reader (Bio Tek).

**Determination of pH-responsive drug release**

The characterized spectrum of RSL3 and diABZi were detected by dispersing them in PBS separately, and recorded from 200-700 nm with Multimode Reader. DP-HBN/RA (5 mg mL^-1^, 1mL) was dialyzed against 20 mL PBS (pH 5.5, 6.5, and 7.4) in dialysis bag (Molecular weight of 3500 Da) in 37 ℃ shaking at 200 rpm. 200 μL of supernatant was collected at different time points in each samples, and quantified them by determining optimal density at 300 nm and 320 nm by Multimode Reader.

**Cell viability assay**

Cell viability was conducted by CCK-8 (Bioss, BA00208) instruction. 3000 4T1 cells per wells were seeded in 96-well plate and incubated for 24 h. Prepared deferent concentration of DP-HBN/RA were added at 12 h before 6 Gy RT. Then washed cells 24 h after RT and added CCK-8 reagent 10 μL per wells with each 100 μL culturing medium. Finally, measuring absorbance density at OD450 after incubation for 30-60 min.

**ROS, mitoROS, and JC-1 detection**

10 μM H2DCFDA (MCE, HY-D0940), 5μM MitoSOX Red (MCE, HY-D1055), and 1ⅹ JC-1 probe (Beyotime, C2003S) were mixed with culturing medium contained pre-treated 4T1 cells at 37 ℃ for 15-30 min. Then washed probes with PBS, and observed with fluorescence microscope.

**GSH, MDA, and iron assay**

The MDA, GSH, and iron level of 4T1 cells were detected with MDA (Solarbio, BC0025) assay kit, GSH assay kit (Solarbio, BC1175), and Cell Ferrous Iron Colorimetric Assay Kit (Elabscience, E-BC-K881-M). Briefly, 4T1 cells were incubated with DP-HBN/RA 12 h before 6 Gy irradiation. Then dissociated cells 24 h after irradiation and cell counting before adding lysis buffer from assay kit, and follow the guidance of assay kits to test cell GSH, MDA, and Fe^2+^ level.

**Immunoblotting**

Lyse pre-treated 4T1 cells with RIPA buffer, PMSF, and Cocktail protease inhibitor (Bioss, C5029, D10411; Servicebio, G2006). Then added 5ⅹ protein loading buffer to degenerate at 100 ℃. Western blotting was performed by incubating with primary antibodies including ACSL4, GPX4, PTGS2 (Biogot, BS7323, BS71431, BS1076), Vinculin, SLC7A11 (ZEN Bio, R26085, R382036), STING, pSTING, cGAS, TBK1, pTBK1, IRF3, pIRF3(Abmart, TD120090, TA7416, PQA3430, T55145, T58364, T55779, TA2436) and secondary antibodies Goat anti-Rabbit IgG (Bioss, bs-0295G-HRP).

**Cell uptake**

4T1 cells were planked in 6-well plates and incubated with FITC-labeled DP-HBN/RA. After incubated with 0, 1, 2, 4, and 6 h, washed 3 times with PBS and stained cells with 4% PFA for 10 min, and then observed under fluorescence microscopy before staining with DAPI (Seven Biothechology, SI103-11).

**Flow cytometry assay**

To detect treated 4T1 cells, harvest 24 h-treated cells with EDTA free lysis to prepare cell suspensions, and wash cells 2 times with PBS, then incubate with different antibodies according to vendor’s protocol (Cell Cycle and Apoptosis Analysis Kit, Annexin V-FITC/PI Apoptosis Detection Kit (MeilunBio, MA0334, MA0220)). To analyze *in vivo* immunological effect, we filtered spleen and lymph node through cell strainers to prepare single-cell suspensions. Cells were stained with 4% PFA after incubates with immunological antibodies.

***In vivo* anti-tumor efficacy experiments**

Female Balb/c mice were inoculated with 4T1 cells with 1ⅹ 10^5^ cells each mice at left leg. When tumor reached 100 mm^3^, the tumor-bearing mice were administrated with PBS, radiation, HBNL, and DP-HBN/RA (2.5 mg kg^-1^) by tail-vein injection. The lung metastasis model was conducted by vein injecetion with 1ⅹ 10^6^ cells each mice. Radiation therapy of 6 Gy was received at Day 2. After 14 d, mice were euthanized and collected tumor, spleen and lymph node for further detection.

**Tolerable dose of DP-HBN/RA in mice**

To determine the tolerable dose of DP-HBN/RA, we employed two endpoints: weight loss and clinical signs. The evaluation of clinical signs was conducted by observing parameters such as activity, appearance, and overall physical condition. The dose range is 0-10 mg kg^-1^, mice were observed daily.

**Long term toxicity assessment**

DP-HBN/RA (5 mg kg^-1^) was injected to healthy mice while control group was injected with PBS. After 28 d, major organs (heart, liver, spleen, lung, and kidney) were selected to visualized with hematoxylin and eosin (H&E) staining.

**Histology and immunohistochemistry**

Xenograft tumor samples were collected and immediately fixed in general tissue staining buffer (Servicebio, G1101) for 24 h. After being washed once with PBS, sequentially dehydrated with graded ethanol (70%, 85%, 95%, and 100%), vitrification by xylene, embedded with paraffin, sectioning. Then H&E staining was proceeded according to Hematoxylin-Eosin (H&E) Stain Kit (Solarbio, G1120) instruction. For immunohistochemical (IHC) staining, after tissue were dehydrated, embedded and sectioned, stoved the slice at 65 ℃ at drying oven. Retrieved antigens by boiled citric acid buffer (Servicebio, G1201) for 15 min. Then processed IHC by UltraSensitive SP IHC kit (MXB biotechnologies, KIT-9710) introduction and DAB reaction kit (Servicebio, G1212). The primary antibody used for IHC was PTGS2, GPX4 (1:100, Biogot, BS1076, BS71431), anti-phospho-histone H2A.X (1:100, Immunoway, YM1429).


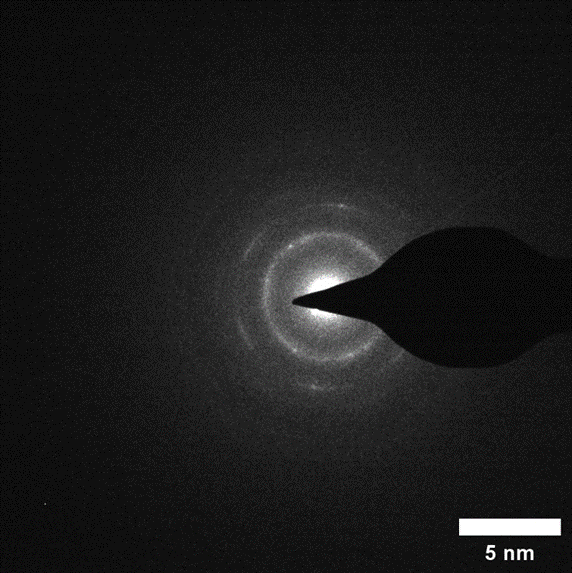


**Figure S1.** Selected area electron diffraction patterns of HBN.


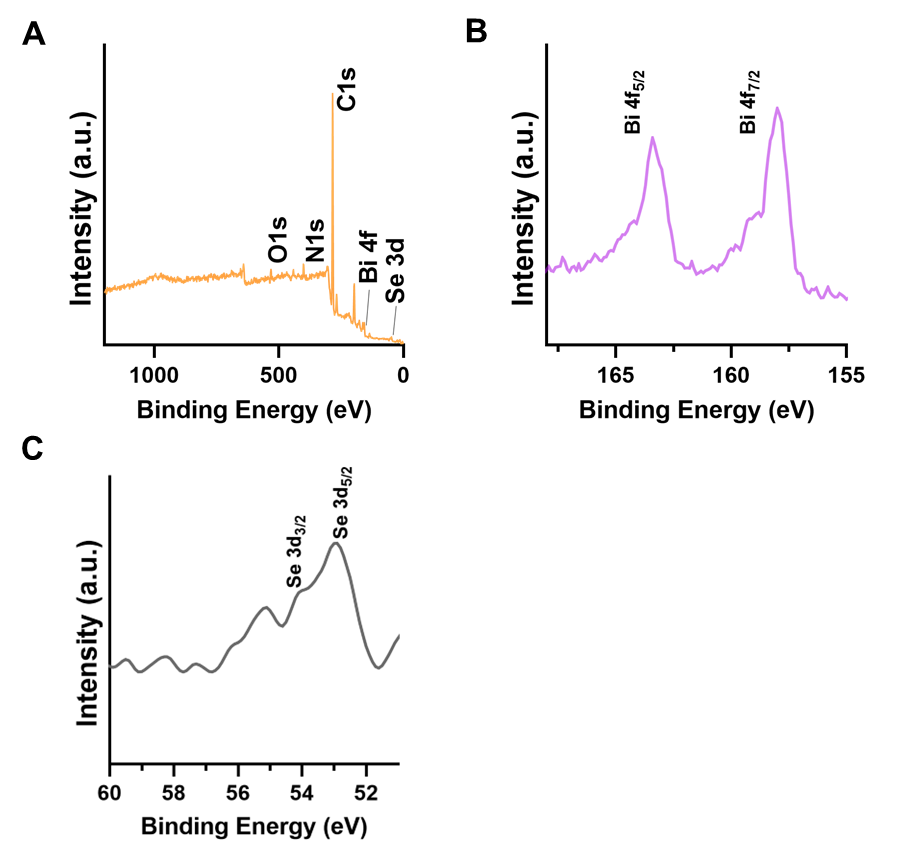


**Figure S2.** XPS spectra of HBN. A) Full spectrum of HBN, B) Bi 4f, C) Se 3d.


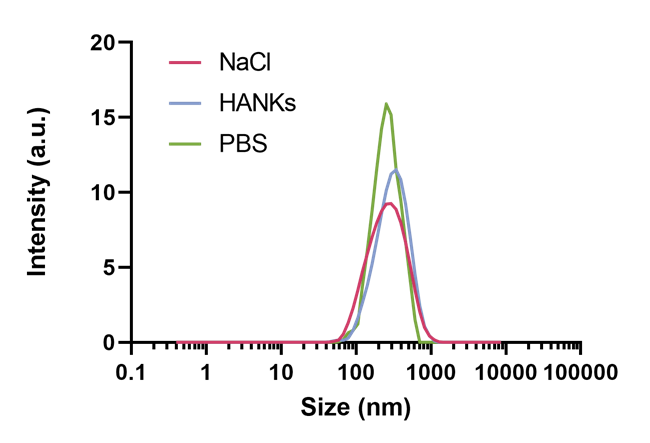


**Figure S3.** DLS of DP-HBN/RA in different human simulated solution.


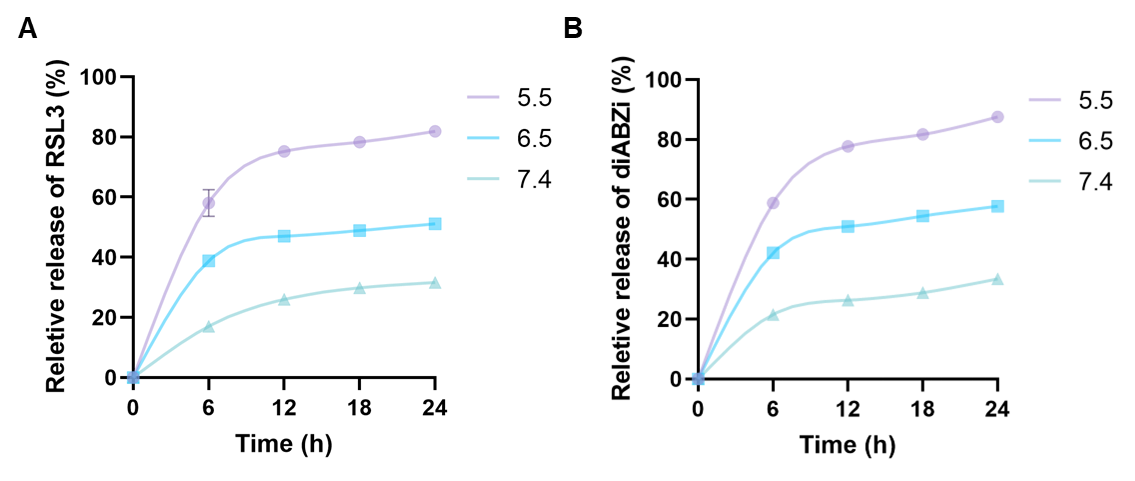


**Figure S4.** A, B) Relative release of RSL3 and diABZi with different time points in different pH conditions.


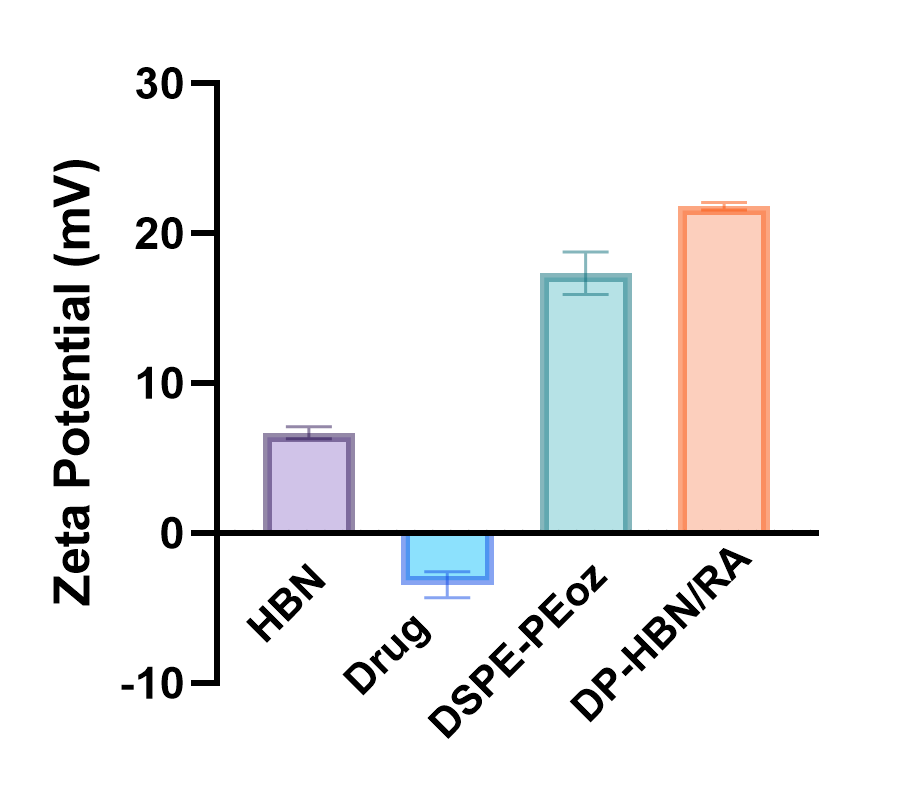


**Figure S5.** Zeta potentials of HBN, Drug (diABZi and RSL3), DSPE-PEoz, and DP-HBN/RA. *n* = 3.


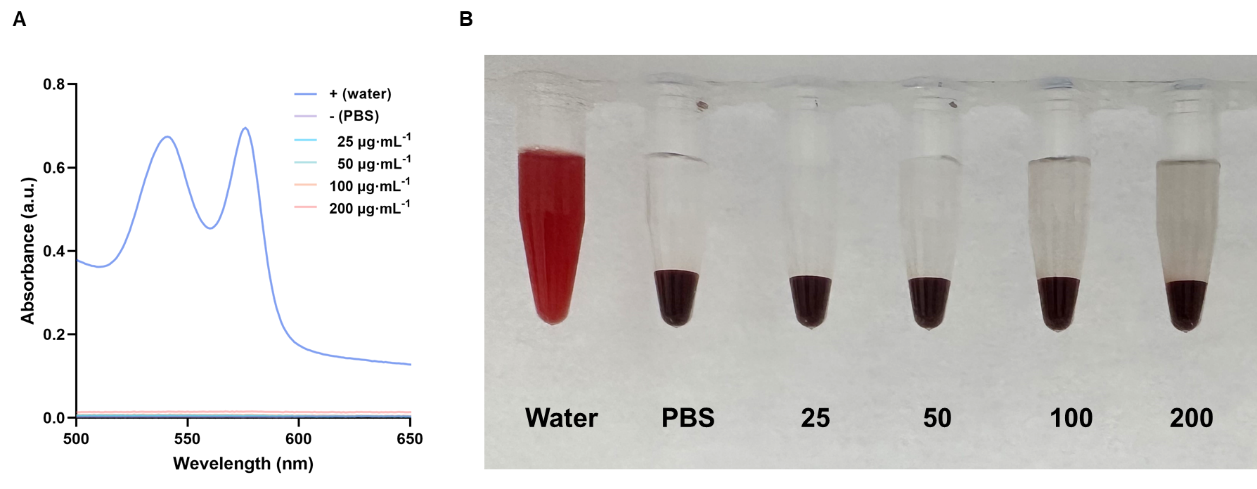


**Figure S6.** A, B) Hemolysis rate of red blood cells incubated with DP-HBN/RA at various concentrations (units: μg mL^-1^).


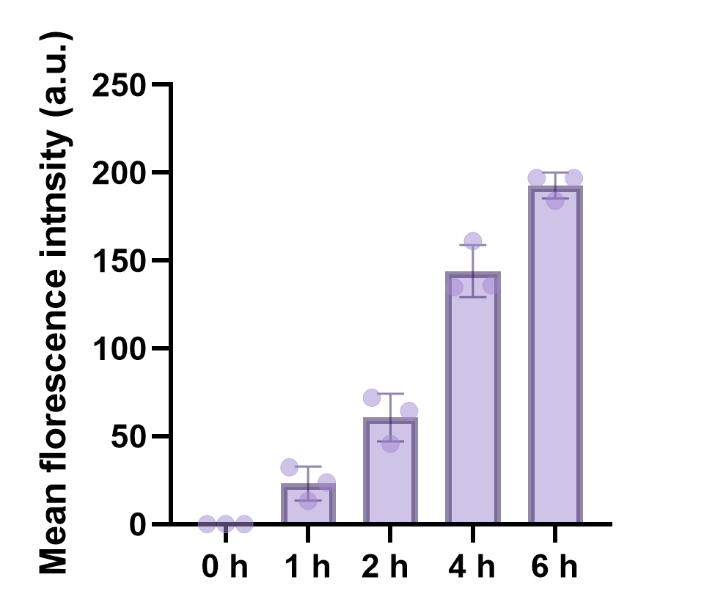


**Figure S7.** Mean florescence intensity of FITC-labeled DP-HBN/RA co-incubate with 4T1 cells.


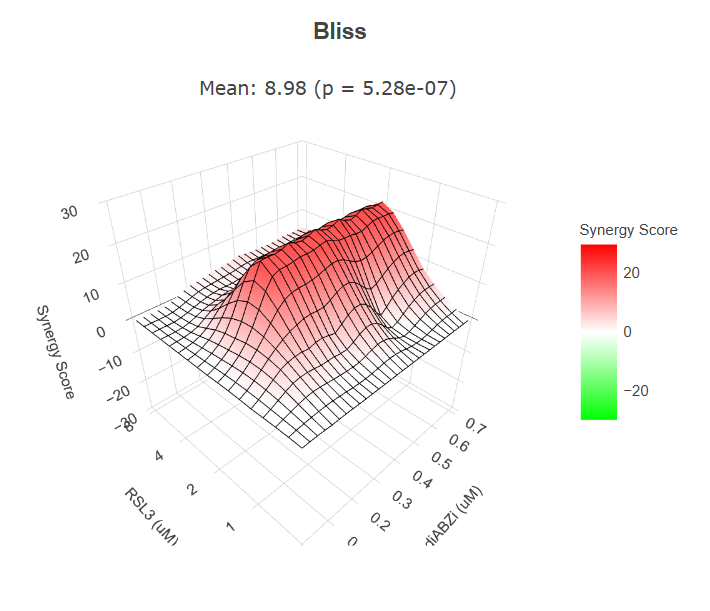


**Figure S8.** The synergistic effect of loaded RSL3 and diABZi on DP-HBN with RT of 6 Gy on 4T1 cells.


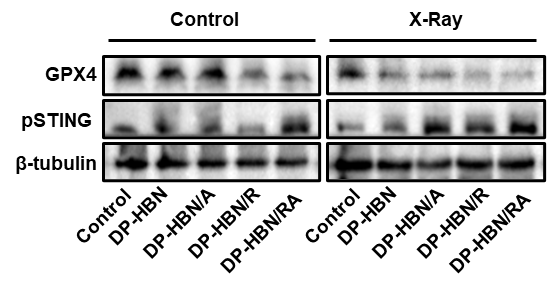


**Figure S9.** Western blotting analysis of GPX4 and pSTING expression in 4T1 cells with different treatments.


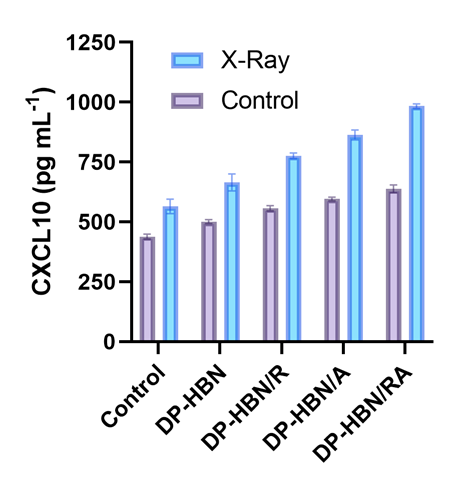


**Figure S10.** Expression of CXCL10 in 4T1 cells with different treatments. Data are presented as mean ± standard deviation.


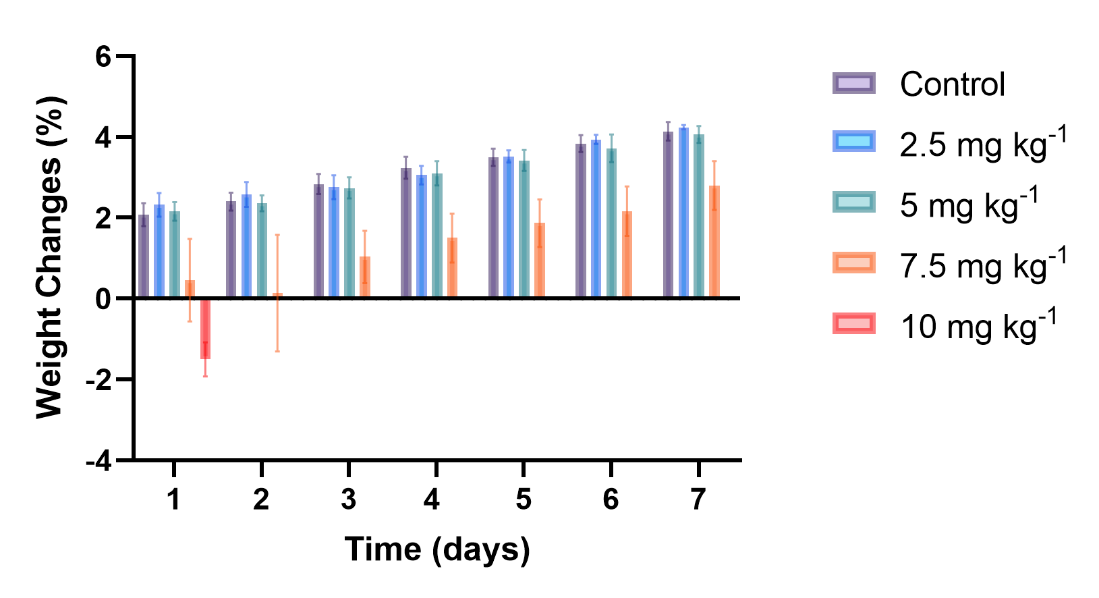


**Figure S11.** The percentage change of body weight of Balb/c mice treated with different.


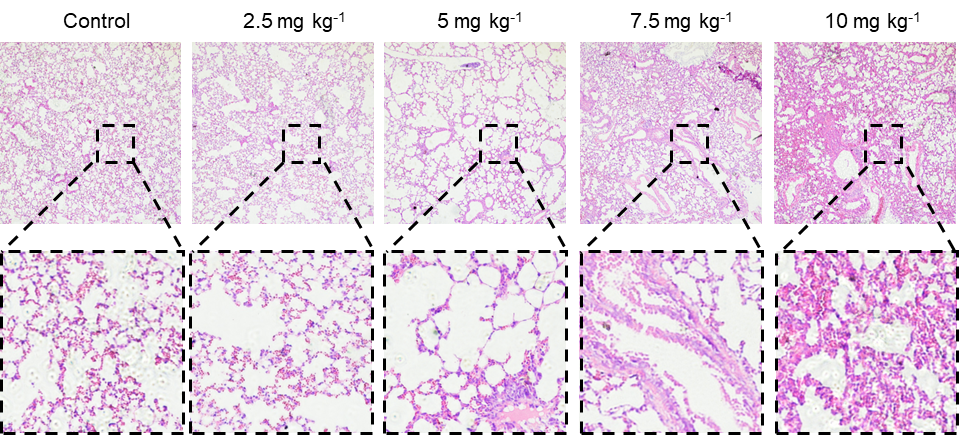


**Figure S12.** H&E staining of lung tissues with different concentration of DP-HBN/RA treated.


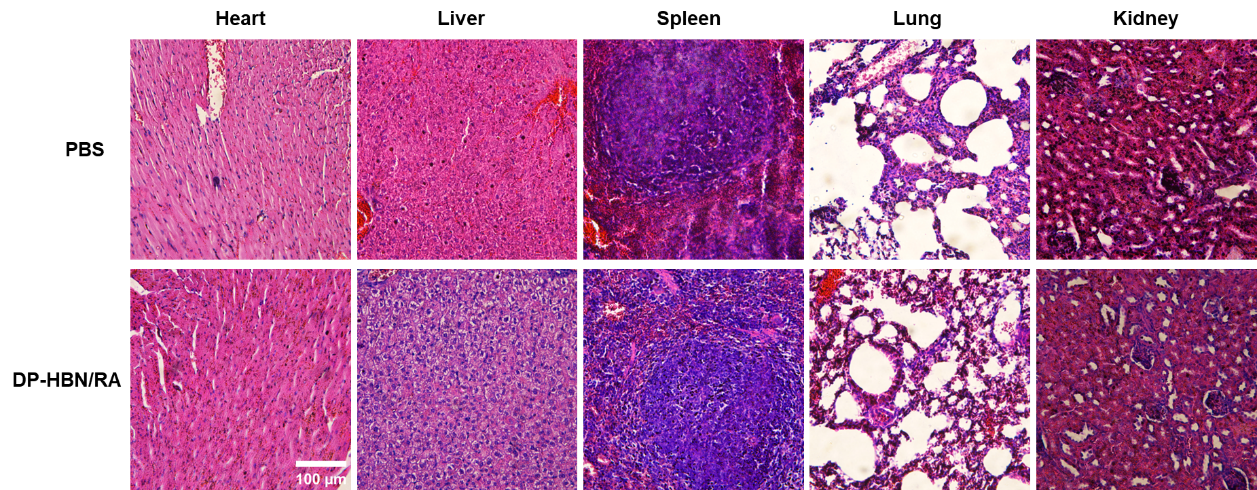


**Figure S13.** H&E staining of major organs after injected DP-HBN/RA for 28 d, scare bar = 100 μm


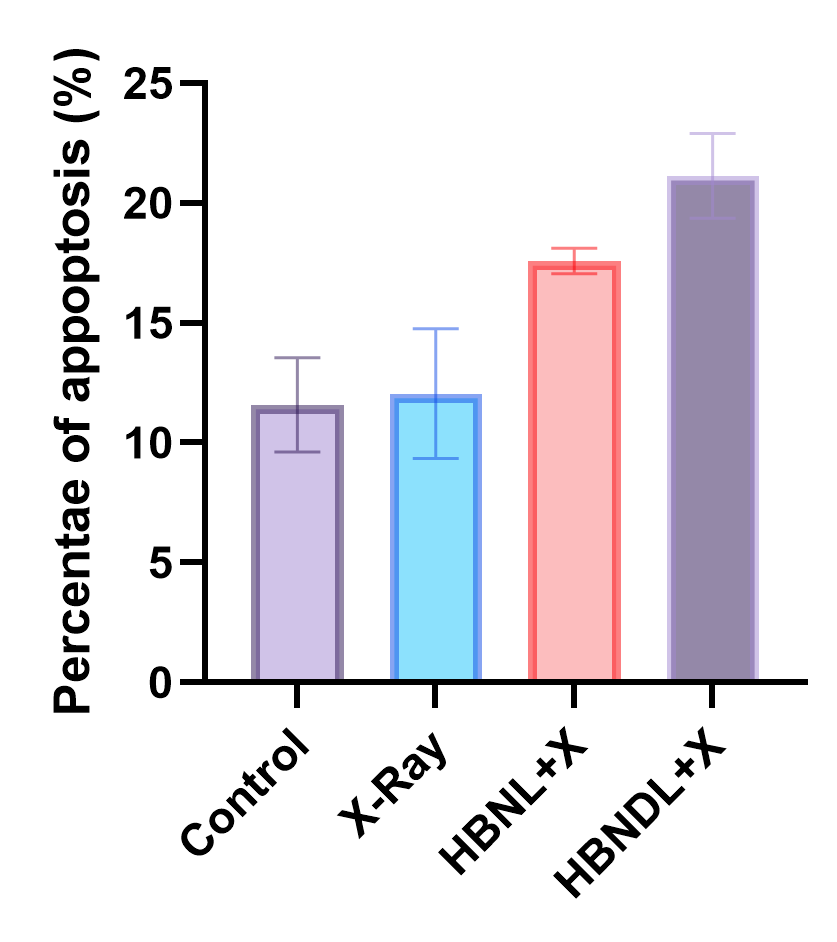


**Figure S14.** Percentage of appoptosis in 4T1 cells.


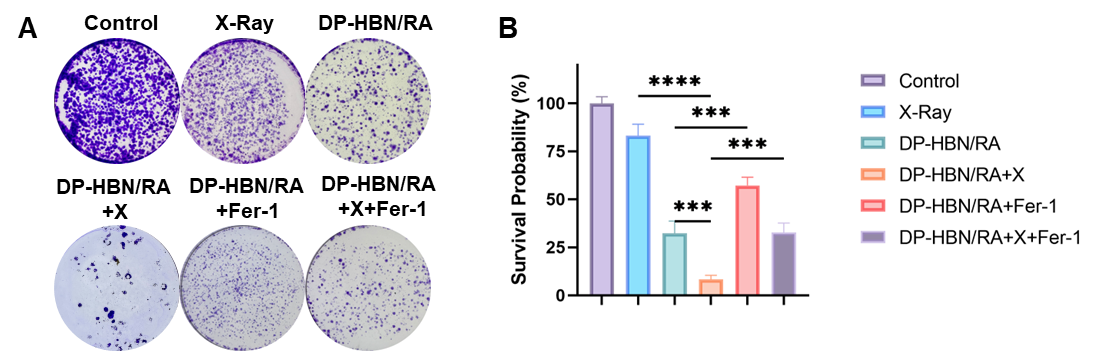


**Figure S15.** Clonogenic survival assay of 4T1 cells. (ANOVA; *** *p* < 0.001and **** *p* < 0.0001).


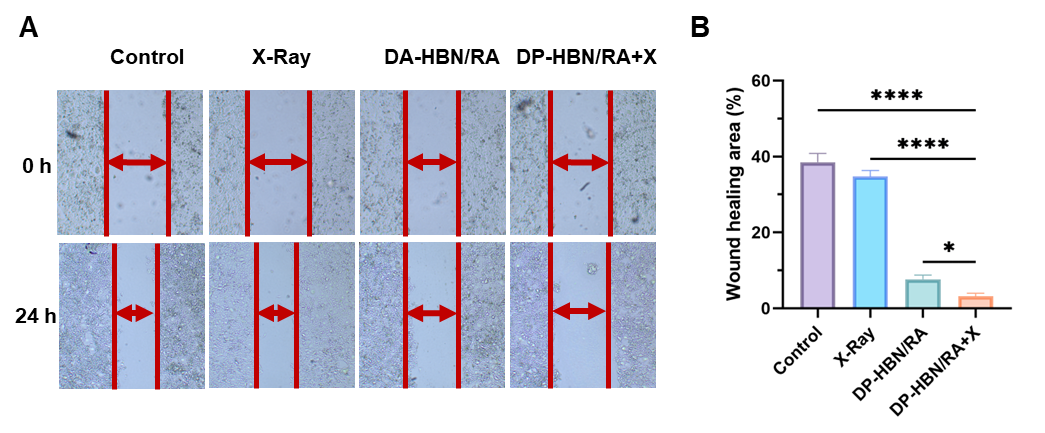


**Figure S16.** Scratching assay of 4T1 cells with different treatments. (ANOVA; * *p* < 0.01 and **** *p* < 0.0001).


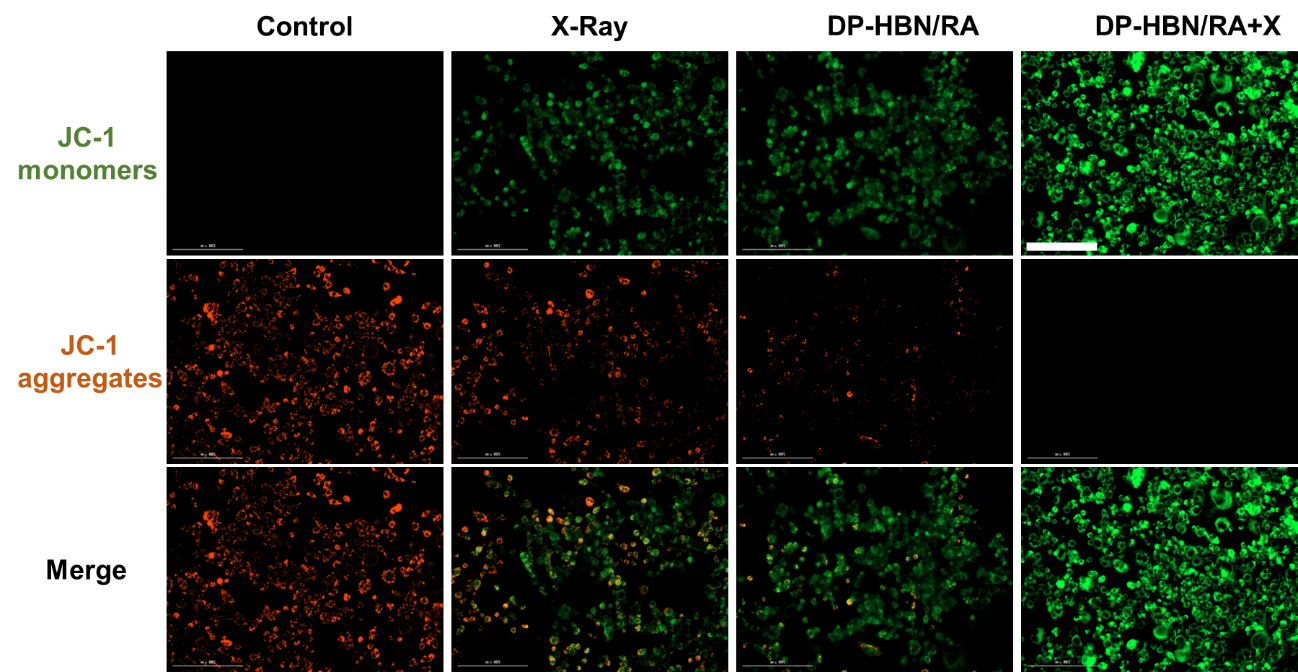


**Figure S17.** JC-1 staining assay with 4T1 cells. scale bar = 100 μm.


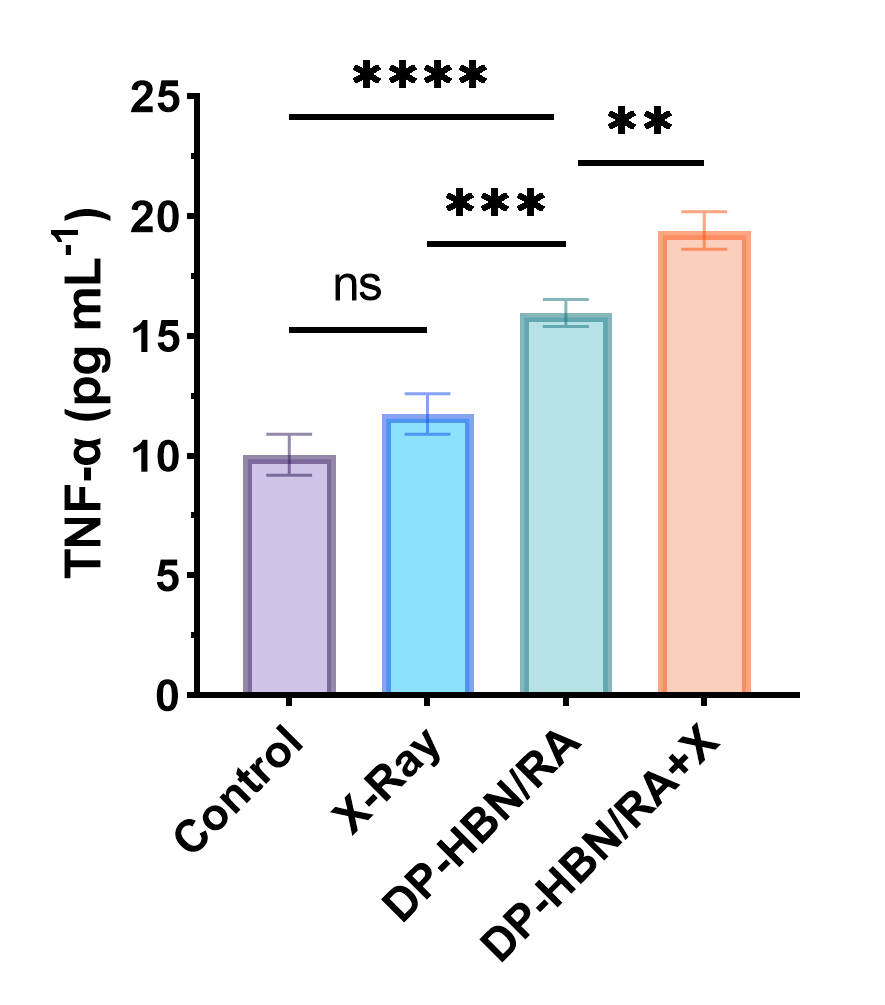


**Figure S18.** Expression of TNF-α in 4T1 cells with different treatments. Data are presented as mean ± standard deviation. (ANOVA; ns for no significance, ** *p* < 0.01, *** *p* < 0.001, and **** *p* < 0.0001).


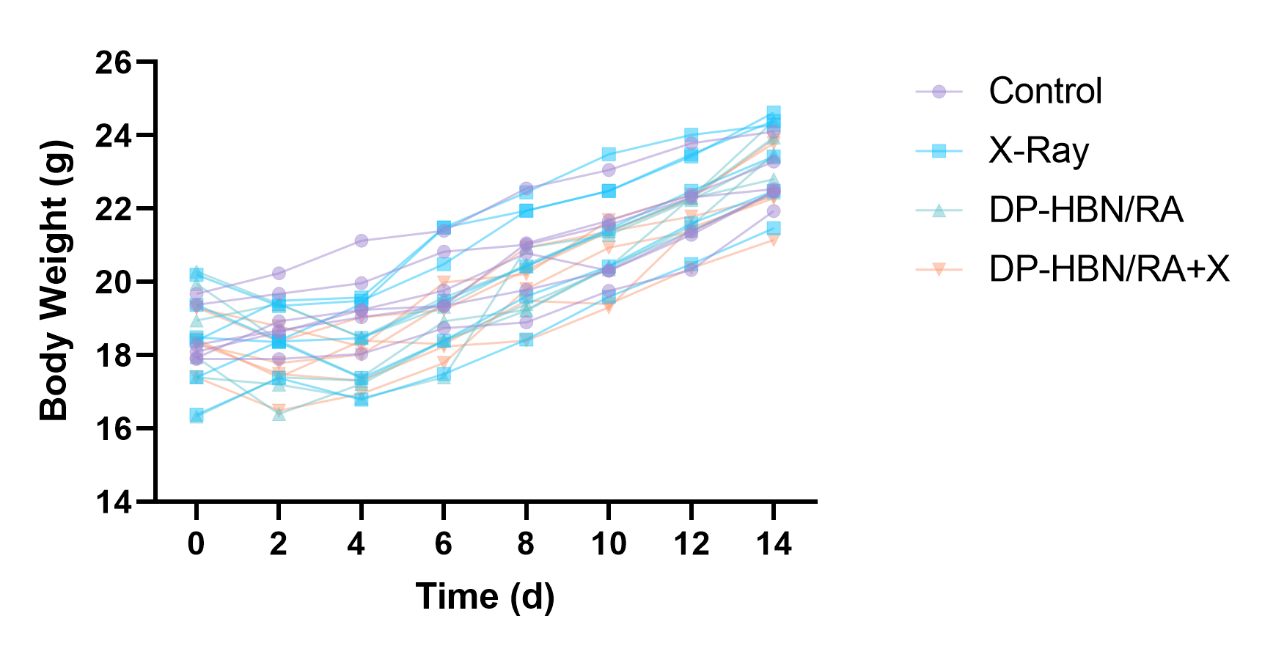


**Figure S19.** Body weight of 4T1-bearing mice with different time points after different treatments.


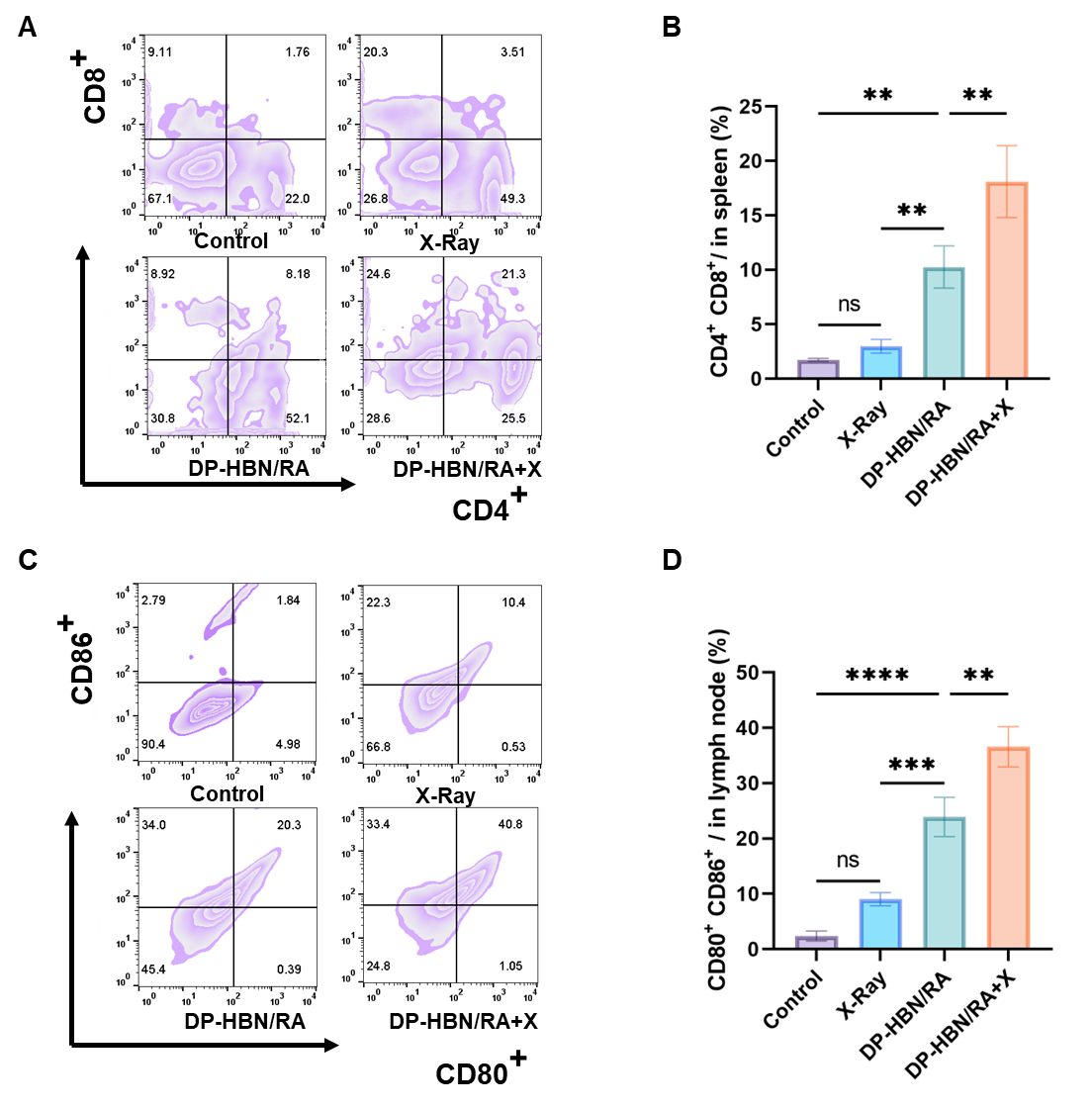


**Figure S20.** Representative flow cytometry plots and quantification in spleen (A and B) and lymph node (C and D) in 4T1-bearing mice. Data are presented as mean ± standard deviation. (ANOVA; ns for no significance, ** *p* < 0.01, *** *p* < 0.001, and **** *p* < 0.0001).


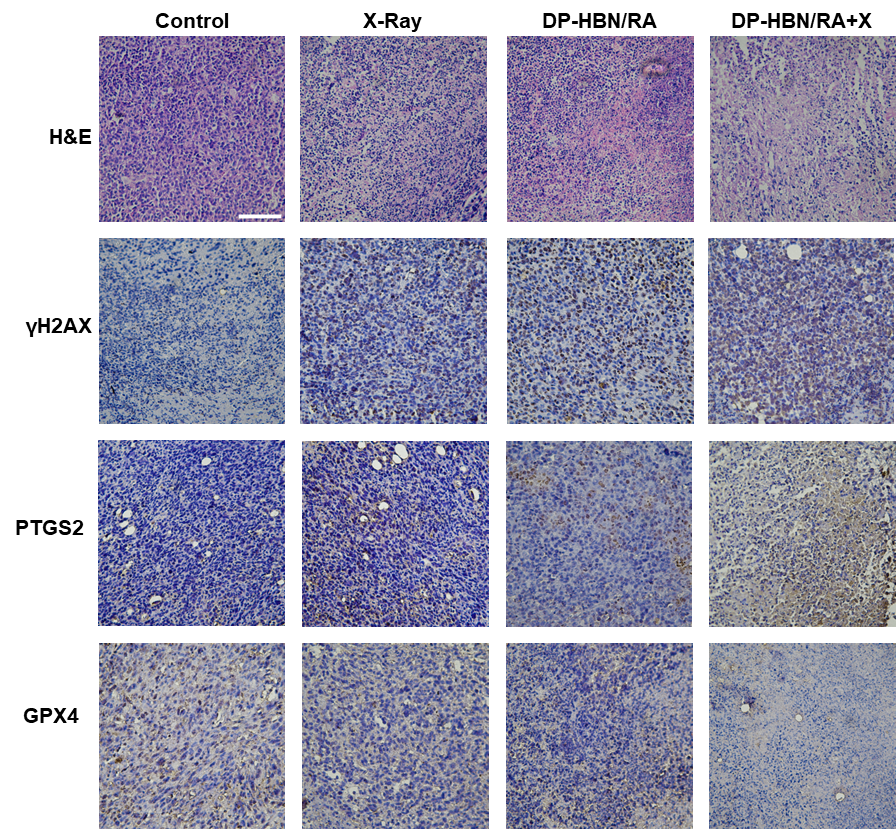


**Figure S21.** H&E staining and ICH (γH2AX, GPX4 and PTGS2) images of 4T1 xenograft tumors with different treatments, scale bar = 100 μm.


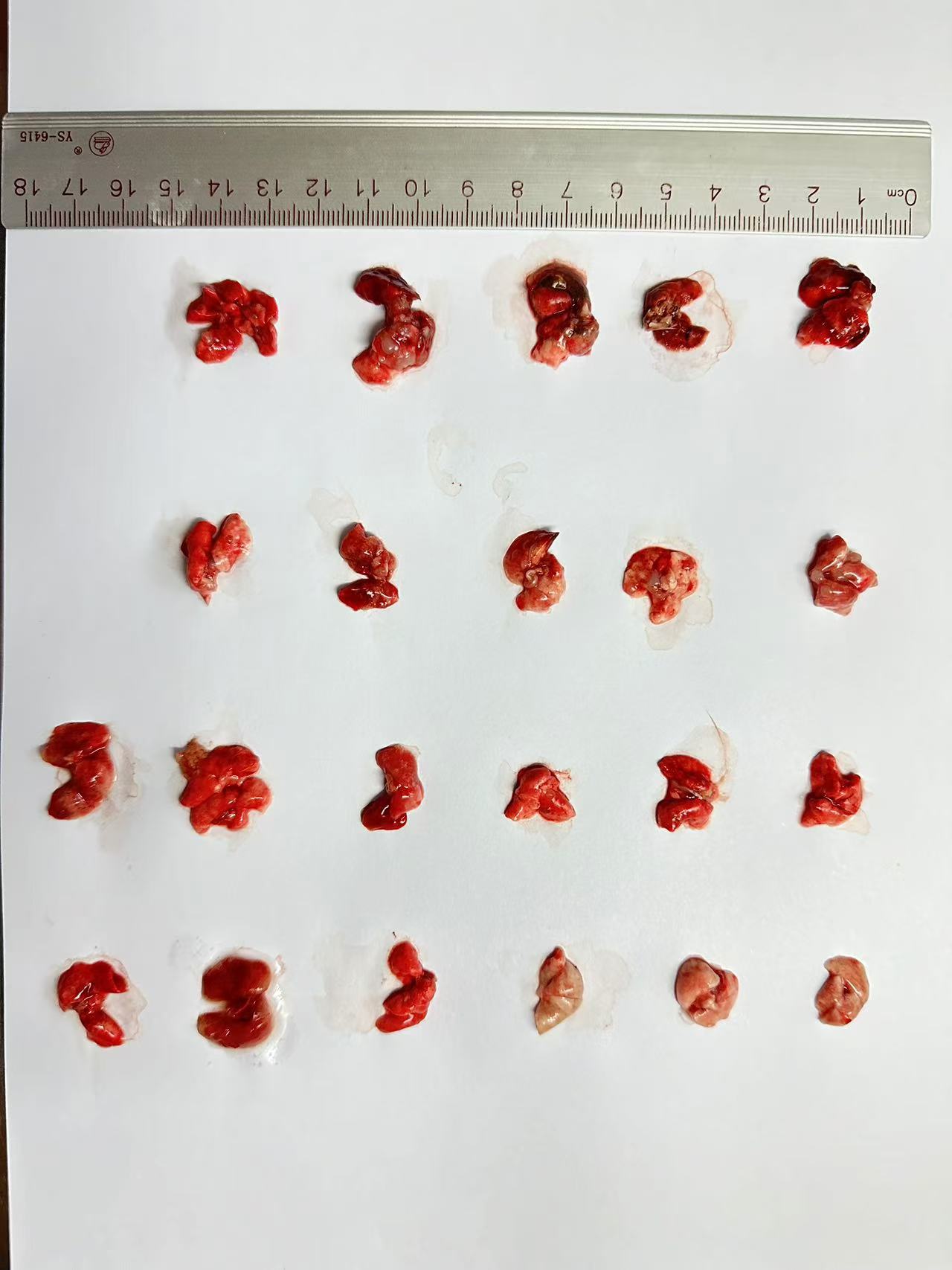


**Figure** **S22.** Photo of lung tissue in 4T1 lung mateastasis mice.


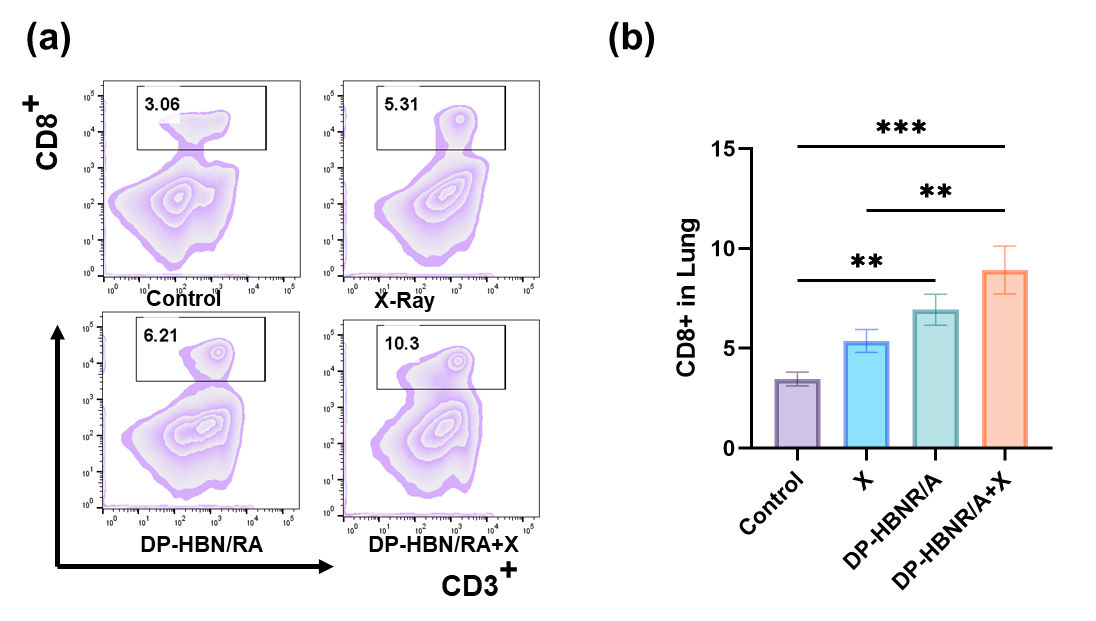


**Figure S23.** Representative flow cytometry plots (a) and quantification (b) of lung tissue in 4T1 lung mateastasis mice. Data are presented as mean ± standard deviation. (ANOVA; ns for no significance, ** *p* < 0.01, *** *p* < 0.001).


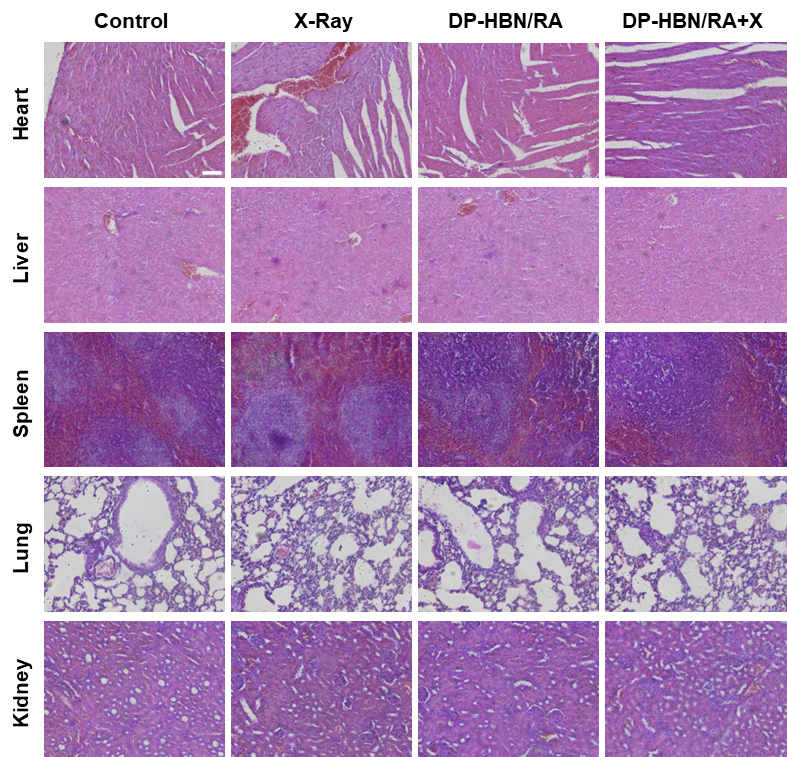


**Figure S24.** H&E staining of major organs with different treatments, scale bar = 100.

**Table S1.** Cage side observation.

|  | Day 1 | Day 2 | Day 4 | Day 4 | Day 5 | Day 6 | Day 7 |
| --- | --- | --- | --- | --- | --- | --- | --- |
| Control | N  (6-6) | N  (6-6) | N  (6-6) | N  (6-6) | N  (6-6) | N  (6-6) | N  (6-6) |
| 2.5 mg kg^-1^ | N  (6-6) | N  (6-6) | N  (6-6) | N  (6-6) | N  (6-6) | N  (6-6) | N  (6-6) |
| 5 mg kg^-1^ | N  (6-6) | N  (6-6) | N  (6-6) | N  (6-6) | N  (6-6) | N  (6-6) | N  (6-6) |
| 7.5 mg kg^-1^ | DAM  (2/6) | DAM  (3/6) | DAM  (3/6) | Dead  (2/6) | DAM  (1/4) | DAM  (1/4) | N  (4/4) |
| 10 mg kg^-1^ | Dead  (4/6) | Dead  (2/2) | - | - | - | - | - |

N: No abnormalities detected

DAM: Decrease Motor Activity
